# Meta-analysis of gene expression profiles of lean and obese PCOS to identify differentially regulated pathways and risk of comorbidities

**DOI:** 10.1016/j.csbj.2020.06.023

**Published:** 2020-06-21

**Authors:** Susan Idicula-Thomas, Ulka Gawde, Sameeksha Bhaye, Khushal Pokar, Gary D. Bader

**Affiliations:** a Biomedical Informatics Centre, Indian Council of Medical Research-National Institute for Research in Reproductive Health, Mumbai 400012, India; b The Donnelly Centre, University of Toronto, Toronto, ON, Canada

**Keywords:** PCOS, Polycystic ovary syndrome, DEGs, Differentially expressed genes, GEO, Gene Expression Omnibus, PCOS, Differential gene expression, Pathway analysis, Enrichment analysis, Comorbidity analysis

## Abstract

Polycystic ovary syndrome (PCOS) is a complex multigenic disorder and women with PCOS suffer from several comorbidities. Although, obesity is a known risk factor for PCOS, the incidence of lean women with PCOS is on the rise. A systematic and comparative study on lean and obese PCOS with respect to genes, pathways and comorbidity analysis has not been attempted so far. Analysis of differentially expressed genes (DEGs) across tissue types for lean and obese PCOS revealed that the majority of them were downregulated for lean and obese PCOS. Ovarian and endometrial tissues shared several commonly dysregulated genes, suggesting shared PCOS pathophysiology mechanisms exist across tissues. Several pathways for cellular homeostasis, such as inflammation and immune response, insulin signaling, steroidogenesis, hormonal and metabolic signaling, regulation of gonadotrophic hormone secretion, cell structure and signaling that are known to be affected in PCOS were found to be enriched in our gene expression analysis of lean and obese PCOS. The gene-disease network is denser for obese PCOS with a higher comorbidity score as compared to lean PCOS.

## Graphical abstract

**Figure:**
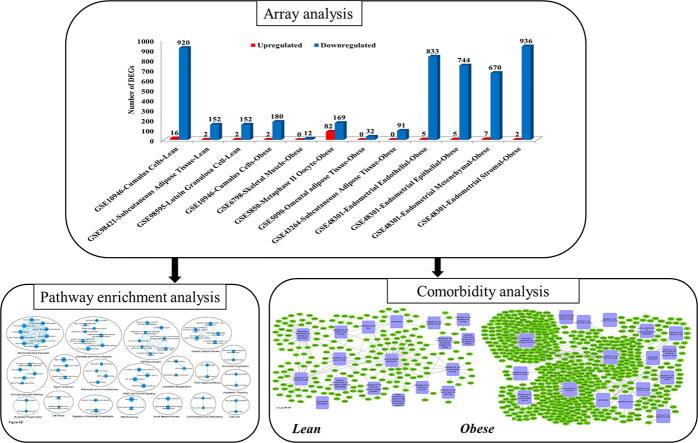

## 1 Introduction

Polycystic ovary syndrome (PCOS) is the most common endocrinological and metabolic disorder reported in women of reproductive age. The cause of the disease can be attributed to genetic and lifestyle factors [1]. The underlying pathophysiology of PCOS, based on our current understanding, can be mainly attributed to elevated LH (Luteinizing Hormone)/FSH (Follicle Stimulating Hormone) ratio and/or insulin [2]. The diagnosis of PCOS is essentially based on three features which include the presence of hyperandrogenism, menstrual irregularity and polycystic ovaries [3].

While obesity is a known risk factor for PCOS, not all women with obesity develop PCOS and not all women with PCOS are obese [4], [5]. Around 30–70% of women, belonging to diverse ethnicities, are affected by PCOS and obesity [6]. On the other hand, 20–50% of women with PCOS are normal weight/lean and the pathophysiology may vary in these two phenotypes [7].

Metabolic syndrome, which is a constellation of conditions such as hypertension, abdominal obesity, insulin resistance and hypercholesterolemia, is commonly seen in women with obesity and PCOS [8], [9]. Dyslipidemia and insulin resistance are more pronounced in obese PCOS as compared to lean PCOS; suggestive of dissimilar metabolic profiles in these phenotypes [4], [10], [11], [12]. For the same reason, the incidence of acanthosis nigricans and impaired lipid profiles and glucose tolerance, which are indicators of insulin resistance, are more widespread in obese PCOS [13].

Altered secretions of adipokines such as adiponectin (*ADIPOQ*), leptin (*LEP*) and resistin (*RETN*) by adipose tissues is one of the important contributory factors to insulin resistance, cardiovascular diseases and metabolic disorders [14], [15]. *ADIPOQ* is downregulated while *LEP* and *RETN* are upregulated in obese conditions [14], [15]. Accordingly, levels of *ADIPOQ* have been found to be lower in obese PCOS as compared to lean PCOS, and levels of *LEP* gene have been reported to be lower in lean PCOS as compared to obese PCOS [15], [16]. Levels of *RETN* were found to be similarly upregulated in obese and lean PCOS cases as compared to controls [14].

Although PCOS and obesity are characterized by increased androgen production, the bioavailable androgen levels are normal in obese non-PCOS cases as compared to PCOS, due to its high clearance rate [5]. Sex hormone binding globulin (*SHBG*) plays a major role in metabolic clearance of free androgens and other hydroxysteroid ligands to the target tissues and liver. Lower serum levels of SHBG in PCOS leads to elevated levels of circulating androgens [17], [18]. The androgen levels are elevated similarly in lean and obese PCOS cases [19]. With respect to hormonal profiles of lean and obese PCOS phenotypes, levels of LH, FSH, LH to FSH ratio, free testosterone, dehydroepiandrosterone (DHEA), anti-müllerian hormone (AMH), estradiol and progesterone are similar in both the phenotypes [19], [20].

The factors associated with PCOS such as anovulation, insulin resistance and altered steroidogenesis are known to increase the risk of cancers in females with PCOS [21], [22], [23]. Amongst the reproductive cancers, clinical studies have reported that women suffering from PCOS have a higher risk of suffering from endometrial cancer [24] followed by ovarian cancer [22]. The mortality rate of ovarian cancer for women who are suffering from obesity and PCOS women is higher as compared to lean women [25]. Although few studies have suggested that the obesity and anovulation in PCOS women can increase the risk of breast cancer [21], [22], the association of breast cancer and PCOS is undecided [26], [27].

The information currently available for lean PCOS is scarce as most of the reported literature is based on patients managed in hospital or fertility clinics, which is known to better represent obese PCOS [19]. Probably for the same reason, there are inconsistencies in the observations from the genetic studies [28]. There is a need to systematically study and compare the gene expression profiles of lean and obese PCOS to gain a more complete understanding of the syndrome.

Here, we identify differentially expressed genes, enriched pathways and associated comorbidities for lean and obese PCOS, based on systematically reviewed and analyzed lean and obese PCOS data from the Gene Expression Omnibus (GEO) [29]. We used a *meta*-analysis approach, where each study containing cases and controls was normalized and analyzed individually to identify differentially expressed genes and enriched pathways and then these results were compared across studies. Information available in literature was used to validate some of the resulting observations. The study has helped to generate novel mechanistic hypotheses for lean and obese phenotypes of PCOS and also to validate existing observations such as higher comorbidity in women who are obese and suffer from PCOS as compared to lean PCOS [19].

## 2 Methods

**Fig. 1:**
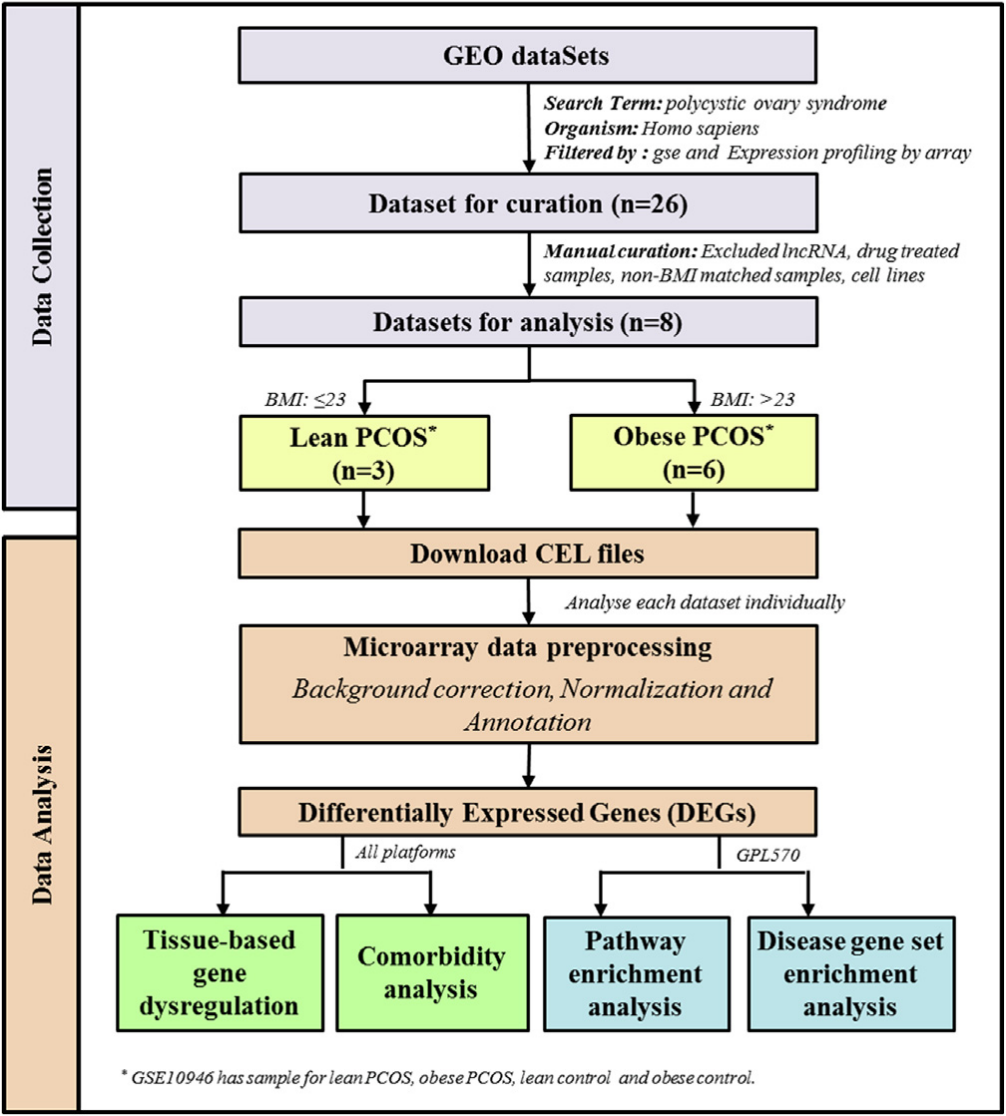
Summary of the workflow adopted in this study.

The workflow adopted in this study is illustrated in Fig. 1.

### 2.1 Data collection and microarray gene expression

The Gene Expression Omnibus (GEO) database was searched on 30th September 2019 to retrieve human microarray gene expression studies on PCOS using the query terms (((“polycystic ovary syndrome”) AND “Homo sapiens”[Organism]) AND gse[Filter]) AND “Expression profiling by array” [Filter]. 26 datasets were identified using this query, which were further manually curated for excluding studies that involved lncRNA, drug-treated samples, cell line studies and non-BMI matched samples. Eight GEO datasets (GSE98421, GSE5850, GSE10946, GSE6798, GSE98595, GSE48301, GSE5090 and GSE43264) qualified for the *meta*-analysis (Table 1, Supplementary Table S1 and Fig. 1). The women in these studies were classified as lean/non-obese (BMI ≤ 23) or obese (BMI > 23) based on their body mass index (BMI). In case of GSE98421, the BMI of the women are not provided in GEO; however the study states that the tissue samples are from lean PCOS patients and hence this study was categorized under lean PCOS.

**Table 1:** Details of the GEO datasets used in the study.

Category	GSE Ids	Cell / tissue type	Sample size		BMI		Platform Ids	Platforms	Age (Years)	No. of Probes/genes
			Control	PCOS	Control	PCOS				
Lean/ Non-obese	GSE98595	Lutein granulosa cells	3	3	22.04	22.39	GPL6244	Affymetrix Human Gene 1.0 ST Array	Control: Average- 27.33, PCOS: Average- 28	33,297
	GSE98421	Subcutaneous adipose tissue	4	4	–	–	GPL570	Affymetrix Human Genome U133 Plus 2.0 Array	–	54,675
	GSE10946	Cumulus cells	6	5	20 ± 4	22 ± 3	GPL570	Affymetrix Human Genome U133 Plus 2.0 Array	Average: 31 (range 29–32)	54,675
Obese	GSE10946	Cumulus cells	5	7	31 ± 4.5	32 ± 4.5	GPL570	Affymetrix Human Genome U133 Plus 2.0 Array	Average: 31 (range 29–32)	54,675
	GSE6798	Skeletal muscle	13	16	34.0 ± 1.8	34.1 ± 1.1	GPL570	Affymetrix Human Genome U133 Plus 2.0 Array	Control: 34.7 ± 2.0, PCOS: 30.8 ± 1.8	54,675
	GSE5850	Metaphase II oocyte	6	6	23.64 ± 1.33	30.98 ± 3.80	GPL570	Affymetrix Human Genome U133 Plus 2.0 Array	Control: 31.30 ± 0.79, PCOS: 30.56 ± 1.41	54,675
	GSE43264	Subcutaneous adipose tissue	7	8	38.25	38.14	GPL15362	NuGO array (human) NuGO_Hs1a520180	–	17,126
	GSE48301	Endometrial epithelial cells (eEP), endothelial cells (eEN), stromal fibroblasts (eSF) and mesenchymal stem cells (eMSC)	15	14	35.73 ± 3.96	≥ 27	GPL6244	Affymetrix Human Gene 1.0 ST Array	Control: 36.50 ± 1.70, PCOS: 30.5 ± 2.1	33,297
	GSE5090	Omental adipose tissue	8	9	52.8 + 5	51.0 + 10.2	GPL96	Affymetrix Human Genome U133A Array	Control: 40.4 ± 3.6, PCOS: 31.6 ± 7.9	22,283

### 2.2 Microarray data pre-processing and identification of differentially expressed genes (DEGs)

The CEL files were retrieved for the selected GEO datasets and each dataset was analyzed following a *meta*-analysis approach, where each case-control study was separately analyzed from raw data to the differentially expressed gene stage, then these DEG lists were compared across studies [30]. In particular, the raw data available in each of the CEL files of the selected GEO datasets were background corrected and quantile normalized. Probe sets were summarized using the Robust Multi-Array Average (RMA) algorithm implemented in the *affy* [31] and *oligo* [32] R packages. Relevant and updated annotations were retrieved for the probesets using the *biomaRt* [33] R Package. Differential gene expression was calculated using the *limma* [34] R package. Statistically significant DEGs were determined based on p-value (p < 0.05) and log fold change (logFC > 2 for upregulated and < -0.5 for downregulated genes). The DEGs were identified with reference to PCOS cases versus controls for each of the GEO datasets.

### 2.3 Analysis using DEGs

The DEGs obtained from each dataset were compared to detect commonly dysregulated genes in PCOS across diverse sample types and GEO platforms. For tissue-based analysis, the aforementioned list of DEGs were clustered based on their tissue source to identify commonly dysregulated genes across the tissue types. The array expression datasets were grouped based on their source, into four tissue types, namely ovarian, endometrial, adipose and skeletal. The ovarian group included metaphase II oocyte, cumulus cells and lutein granulosa cells. The endometrial group included cell types of epithelial, endothelial, stromal fibroblasts and mesenchymal stem cells. The adipose group had subcutaneous adipose tissue and omental adipose tissue. The skeletal group had skeletal muscle tissue.

### 2.4 Pathway enrichment analysis

Pathway enrichment analysis was performed using the Gene Set Enrichment Analysis (GSEA) method. The Java desktop application of GSEA (v. 3.0) developed by Broad Institute was used to identify statistically enriched gene sets and pathways in each of the datasets [35]. The Bader lab human gene set database containing updated information collected from various pathway databases such as GO [36], Reactome [37], KEGG [38] and MsigDB [39], excluding annotations that have evidence code IEA (inferred from electronic annotation), ND (no biological data available) and RCA (inferred from reviewed computational analysis) were used for GSEA analysis [40]. The “Max” size was set at 200 and “Min” size was set at 10 in order to remove the “too general” and “too specific” gene sets and pathways, respectively. The number of permutations was set to 2000. The analysis was performed using the weighted enrichment statistic, using the default weight set to p = 1.

The GSEA output and normalized expression data were used to perform Enrichment analysis. The Enrichment Map Analysis Pipeline [41] in Cytoscape version 3.6.1 [42] was used to visualize the pathway enrichment analysis results. All the parameters were set to their defaults. FDR q-value and p-value cutoff were set at 0.1 and 1.0, respectively. For all datasets of GPL570, individual networks were created. A master network was created using all tissue types of GPL570 only, except metaphase II oocyte (as oocyte may have distinct cellular events and metabolic pathways) for identifying the common and unique enriched pathways in lean and obese PCOS. The AutoAnnotate [43] Cytoscape app was used to identify clusters present in the enrichment map for grouping redundant pathways and ease of interpretation.

### 2.5 Comorbidities and disease distribution in lean and obese PCOS

The KEGG disease database (Release 88.2) was used to obtain information for human diseases and its associated genes. The diseases were categorized as per International Classification of Diseases 11th Revision (ICD-11). The KEGG gene and disease IDs were used for mapping genes with the highest level of ICD11 classification. The DEGs were further mapped to this data to obtain the gene-disease association score (GDS) for PCOS. For each ICD-11 category, GDS was calculated as below

$$\mathrm{GDS}=\frac{\mathrm{Number}\;\mathrm{of}\;\mathrm{DEGs}\;\mathrm{from}\;\mathrm{meta}\mathrm{analysis}\;\mathrm{mapped}\;\mathrm{to}\;\mathrm{the}\;\mathrm{disease}}{\mathrm{Total}\;\mathrm{number}\;\mathrm{of}\;\mathrm{genes}\;\mathrm{mapped}\;\mathrm{to}\;\mathrm{the}\;\mathrm{disease}}\times 100$$

Gene-disease association was further used to construct gene-disease networks for lean and obese PCOS.

### 2.6 Gene set variation analysis (GSVA)

The R packages *GSVA* [44] and *complexheatmap* [45] were used to generate a heatmap displaying the variation of gene sets in different tissues of women with PCOS. The gene expression matrix (logFC values) was analyzed by GSVA using gene set wherein each gene set contains a list of genes associated with diseases classified by ICD-11 codes from the KEGG database. GSVA was performed to understand the regulation of genes across the ovarian tissue types for lean and obese PCOS (GPL570 platform only) and its impact on reproductive and endocrine diseases.

## 3 Results

### 3.1 The majority of differentially expressed genes in PCOS are downregulated

**Fig. 2:**
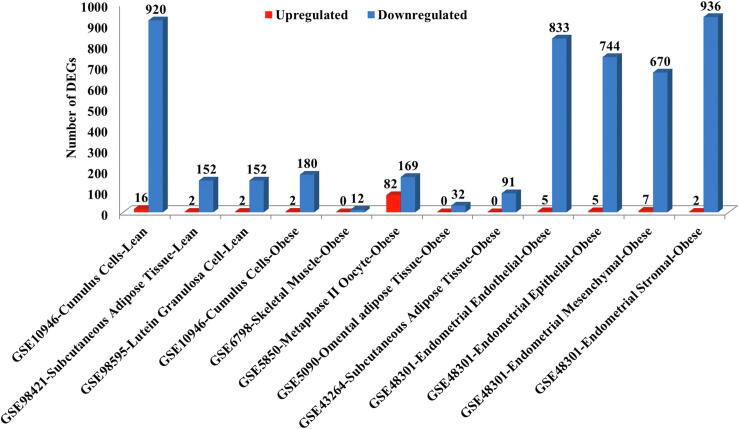
DEGs across all tissue types. For each dataset (x-axis), DEGs were identified based on p-value <0.05 and logFC >2 for upregulation (red bars) and <−0.5 for downregulation (blue bars). (For interpretation of the references to colour in this figure legend, the reader is referred to the web version of this article.)

A total of 5014 (unique = 4224) statistically significant DEGs were identified by analyzing the eight GEO datasets individually (see Method section 2.2, Supplementary Table S2). Of these, 123 (unique = 96) genes were upregulated and 4891 (unique = 4101) genes were downregulated (Supplementary Table S3). Regardless of tissue type and phenotype, the majority of the genes were downregulated in PCOS. Endometrial cells of obese PCOS and cumulus cells of lean PCOS displayed the highest number of dysregulated genes (Fig. 2). Seven (*ETV3, GABPB1, ELF3, GABPA, ELF1, ELF4* and *SRF*) genes were identified to encode transcription factors using the iRegulon Cytoscape app [46] (Supplementary Table S4). Of the 4224 unique DEGs, the association of 136 genes with PCOS has been established in the literature. The links to the relevant publications can be viewed under the “Literature Evidence” column in Supplementary Table S2.

### 3.2 Commonly dysregulated genes are identified in lean PCOS

Array analysis of lean and obese PCOS, revealed that there were no commonly dysregulated genes across the platforms and tissue sources. Seven genes (*PRRT1, SLITRK4, CRHBP, HAPLN1, SRGN, EREG* and *WNT5A*) were found to be commonly dysregulated when analysis was restricted to two tissues (cumulus cells and subcutaneous adipose tissue) using GPL570 platform for lean PCOS (Supplementary Table S5).

*WNT5A* participates in the WNT signaling pathway that is associated with tissue development process and inflammatory response [47]. *WNT5A* has been reported to be overexpressed in the granulosa cells of lean women with PCOS through qPCR studies [48] and this is in agreement with our observation of upregulation of *WNT5A* expression in cumulus granulosa cells of lean PCOS (GSE10946) [49]. The association and the regulation status of the other six genes in PCOS is not well-studied and it would be worthwhile to investigate the role of these genes in the pathophysiology of PCOS.

### 3.3 Tissue-based gene dysregulation in PCOS

**Fig. 3:**
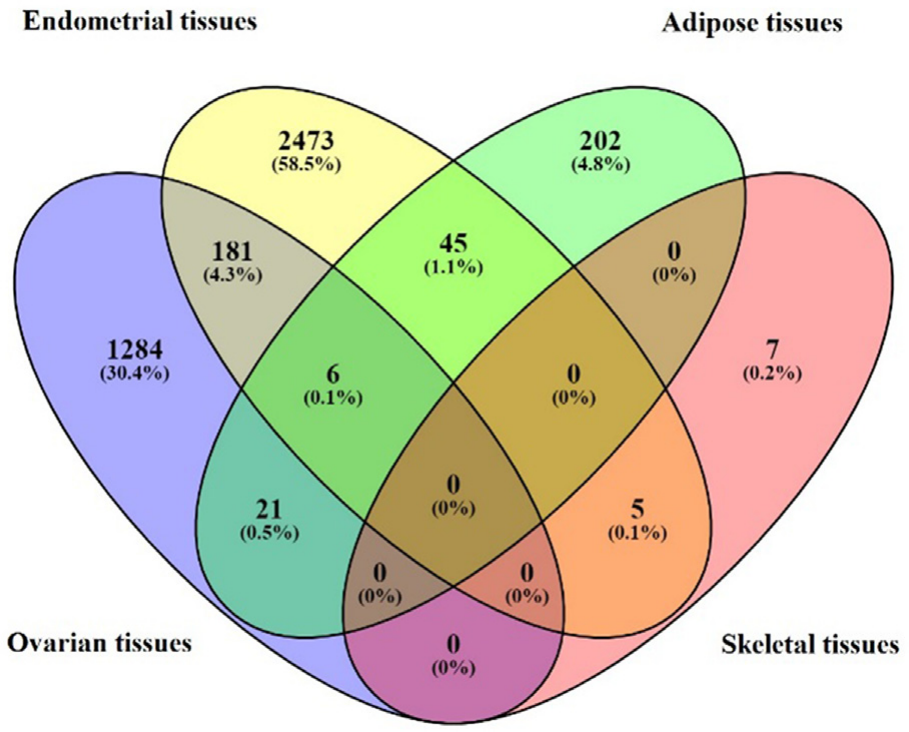
Tissue-based distribution of DEGs in PCOS. Endometrial tissues presented the maximum number of DEGs. Ovarian and endometrial tissues shared the maximum number of common DEGs.

Of the 4224 identified DEGs, 1284 were exclusive to ovarian tissue, 2473 were exclusive to endometrial tissue, 202 were exclusive to adipose tissue and 7 were exclusive to skeletal tissue. Six genes (*GPX7, SERPINI1, TMEM256, SVIP, MAT2A* and *SRGN*) were commonly dysregulated in ovarian, endometrial and adipose tissues. Apart from these six genes, ovarian and endometrial tissues shared 181 (4.3%); ovarian and adipose tissues shared 21 (0.5%); adipose and endometrial tissues shared 45 (1.1%); endometrial and skeletal tissues shared 5 (0.1%) commonly dysregulated genes (Fig. 3, Supplementary Table S6).

### 3.4 Biological pathways enriched in lean and obese PCOS

**Fig. 4:**
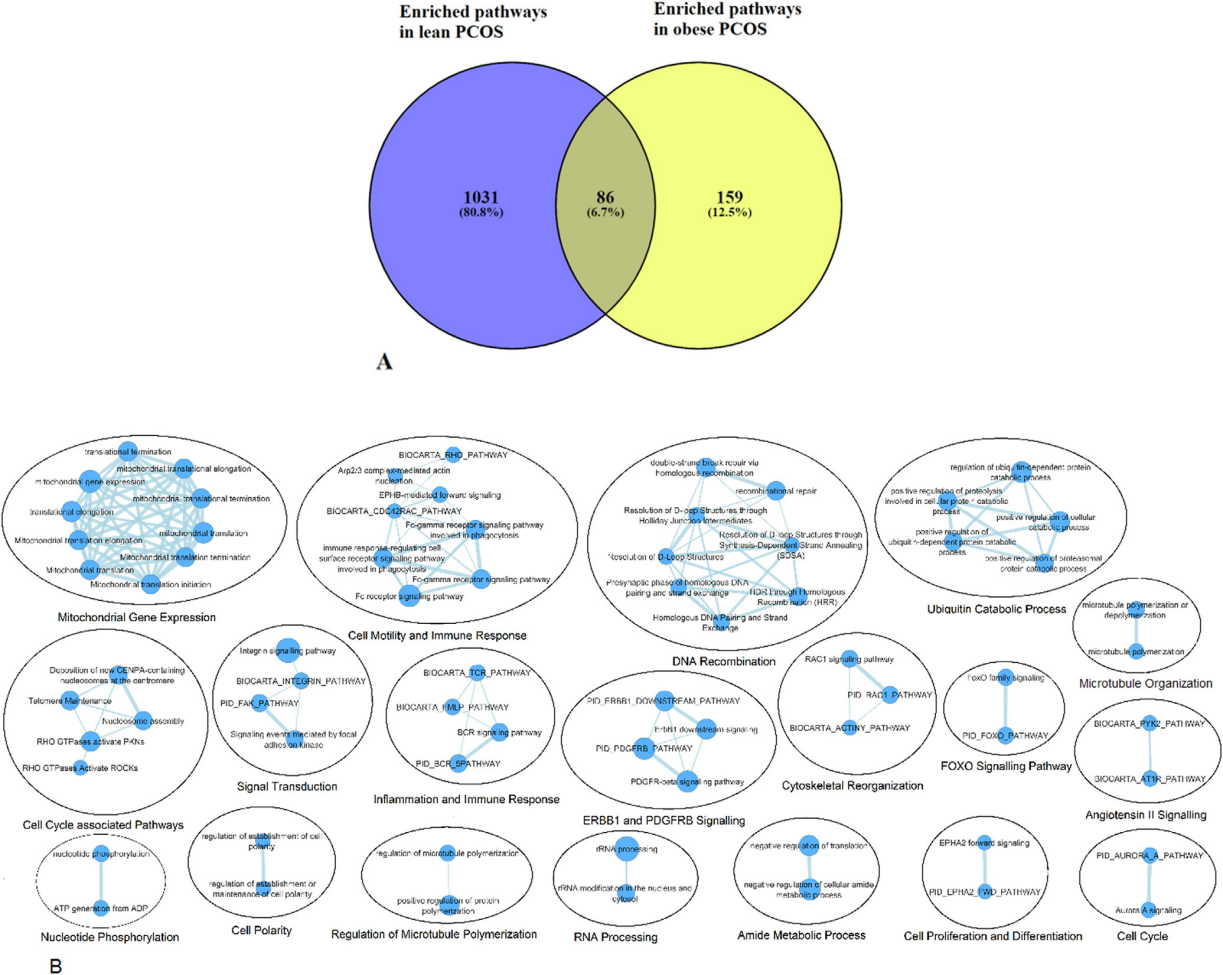
A) Pathway analysis of lean and obese PCOS. 86 pathway gene sets are commonly enriched in lean and obese PCOS. B) Biological pathways enriched in lean and obese PCOS. Nodes represent pathway gene sets. Size of the node is indicative of the number of genes involved in the pathway. An edge between nodes represents shared genes between the pathways. Pathways are grouped by similarity. The analysis was restricted to commonly dysregulated genes for GPL570.

The pathway gene sets that were found to be enriched across all tissues for lean and obese PCOS from GPL570 are listed in Supplementary Table S7. Comparison of the enriched pathway gene sets identified from studies on GPL570 (GSE10946 lean and obese, GSE98421 lean, GSE6798 obese) revealed that there were 86 pathway gene sets (6.7%) common for lean and obese PCOS, 1031 pathway gene sets (80.8%) unique to lean PCOS and 159 pathway gene sets (12.5%) unique to obese PCOS cases (Fig. 4A, Supplementary Fig. S1).

#### 3.4.1 Diverse pathways are commonly enriched across tissue types

Biological pathway gene sets that are enriched based on the commonly dysregulated genes in lean and obese PCOS included mitochondrial gene expression, cell migration, DNA recombination, ubiquitin catabolic process, cell cycle associated pathways, inflammation and immune response, cell growth and development, cell adhesion and signal transduction, cytoskeletal reorganization, cell cycle progression, nucleotide phosphorylation, regulation of ion levels, FOXO signaling, cell adhesion and differentiation, microtubule organization, negative regulation of amide metabolic process, RNA processing, cell polarity regulation and regulation of microtubule polymerization (Fig. 4B).

#### 3.4.2 Differentially regulated biological pathways group into four major themes

The pathways, obtained from enrichment analysis of differentially regulated genes in lean and obese PCOS, were clustered based on functional themes. Four themes were observed: (i) Cell-motility and immune response; (ii) FAK-related; (iii) ERBB1 and PDGFRB signaling; and (iv) Mitochondrial gene expression. Pathway gene sets involved in ‘Cell-motility and Immune response’, ‘FAK-related’ and ‘ERBB1 and PDGFRB signaling’ were downregulated in lean PCOS and upregulated in obese PCOS. Pathway gene sets involved in ‘Mitochondrial gene expression’ were upregulated in lean PCOS and downregulated in obese PCOS (Table 2).

**Table 2:** Differentially regulated biological pathways in lean and obese PCOS

Cluster	Lean PCOS	Obese PCOS
Cell-motility and immune response		
FAK-related		
ERBB1 and PDGFRB signaling		
Mitochondrial gene expression *		

* Pathway names are redundant due to the use of multiple database sources. For example, mitochondrial translation involves 111 genes from the GOBP database and Mitochondrial translation involves 93 genes from the Reactome database. Each node is segmented into two halves representing the two constituent GEO datasets (GSE10946 and GSE98421 for lean PCOS; GSE10946 and GSE6798 for obese PCOS) of GPL570 and is colored based on the normalized enrichment scores (NES) values obtained from GSEA analysis. Red and blue represents upregulation and downregulation, respectively.

### 3.5 Differentially expressed genes in PCOS are also linked to developmental, metabolic and nervous system diseases

**Fig. 5:**
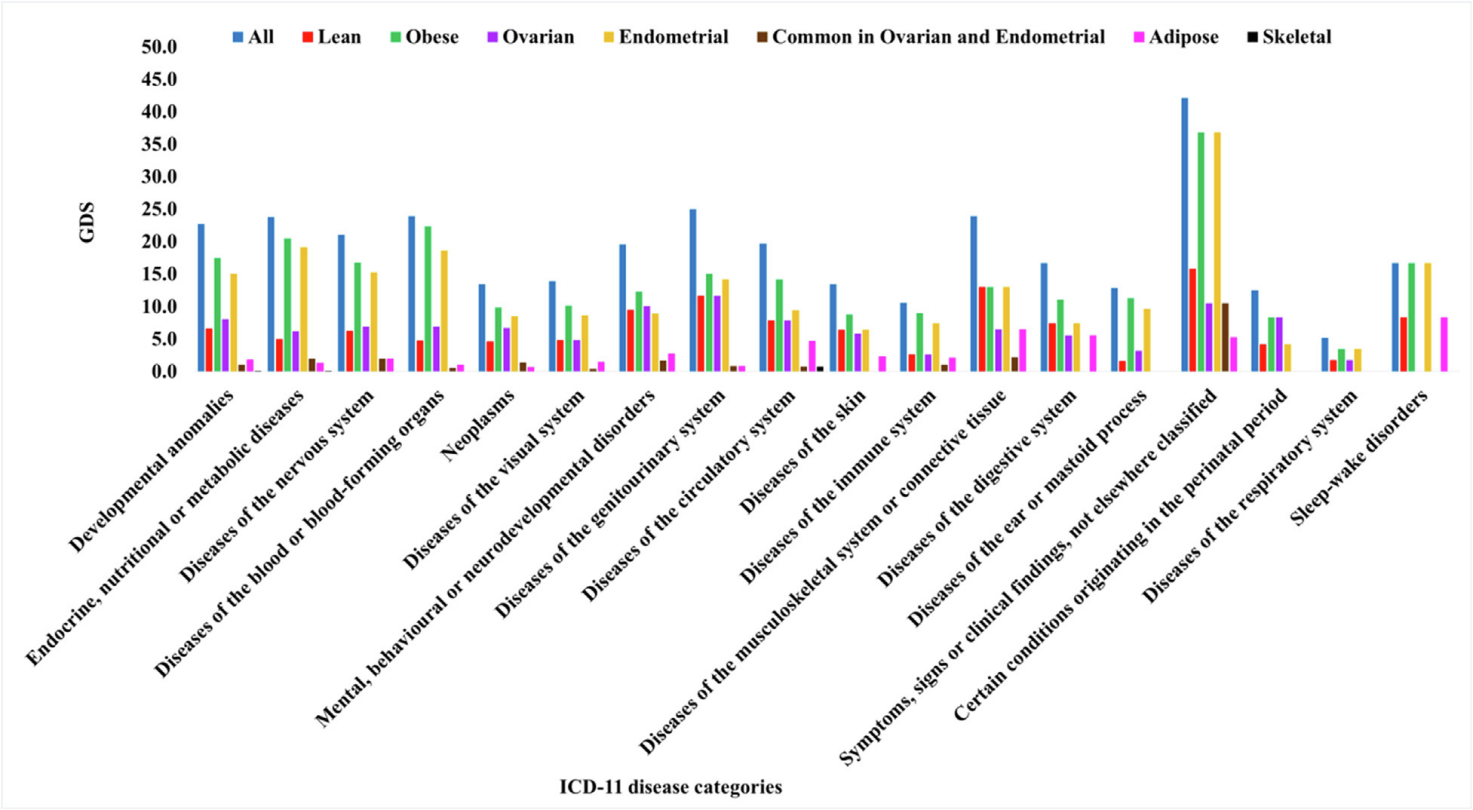
GDS analysis for lean and obese PCOS. Blue bars indicate the percentage of DEGs mapped to the disease groups across all tissue types and array platforms. Red and green bars indicate the percentage of DEGs mapped to the disease based on lean and obese PCOS datasets respectively. The purple, orange, brown, pink and black bars indicate the percentage of DEGs mapped to the disease based on analysis of ovarian, endometrial, ovarian + endometrial, adipose and skeletal tissues respectively. (For interpretation of the references to colour in this figure legend, the reader is referred to the web version of this article.)

The identified DEGs were mapped to genes associated with the ICD-11 disease categories (Supplementary Fig. S2). The maximum number of DEGs were mapped to developmental anomalies (222 genes), followed by disorders of the metabolic (192 genes) and nervous system (127 genes). It is interesting to note that 37 DEGs mapped to disorders of the visual system (Supplementary Table S8). This list included genes such as *CFH* and *CYP1B1* which are well studied for its role in eye diseases [50]. There are very few published reports on association of PCOS and disorders of visual system [51]. It would be worthwhile to evaluate the expression of these DEGs in ocular tissues to confirm the comorbidity hypothesis. Another interesting observation was that 8 DEGs, including *FN1, ACTN4* and *TRPC6* mapped to the pathological condition of the glomerulus. It is noteworthy that DEGs identified from GEO datasets of obese PCOS and endometrial tissues displayed maximum GDS (Fig. 5).

#### 3.5.1 Phenotype-specific gene-disease network

**Fig. 6:**
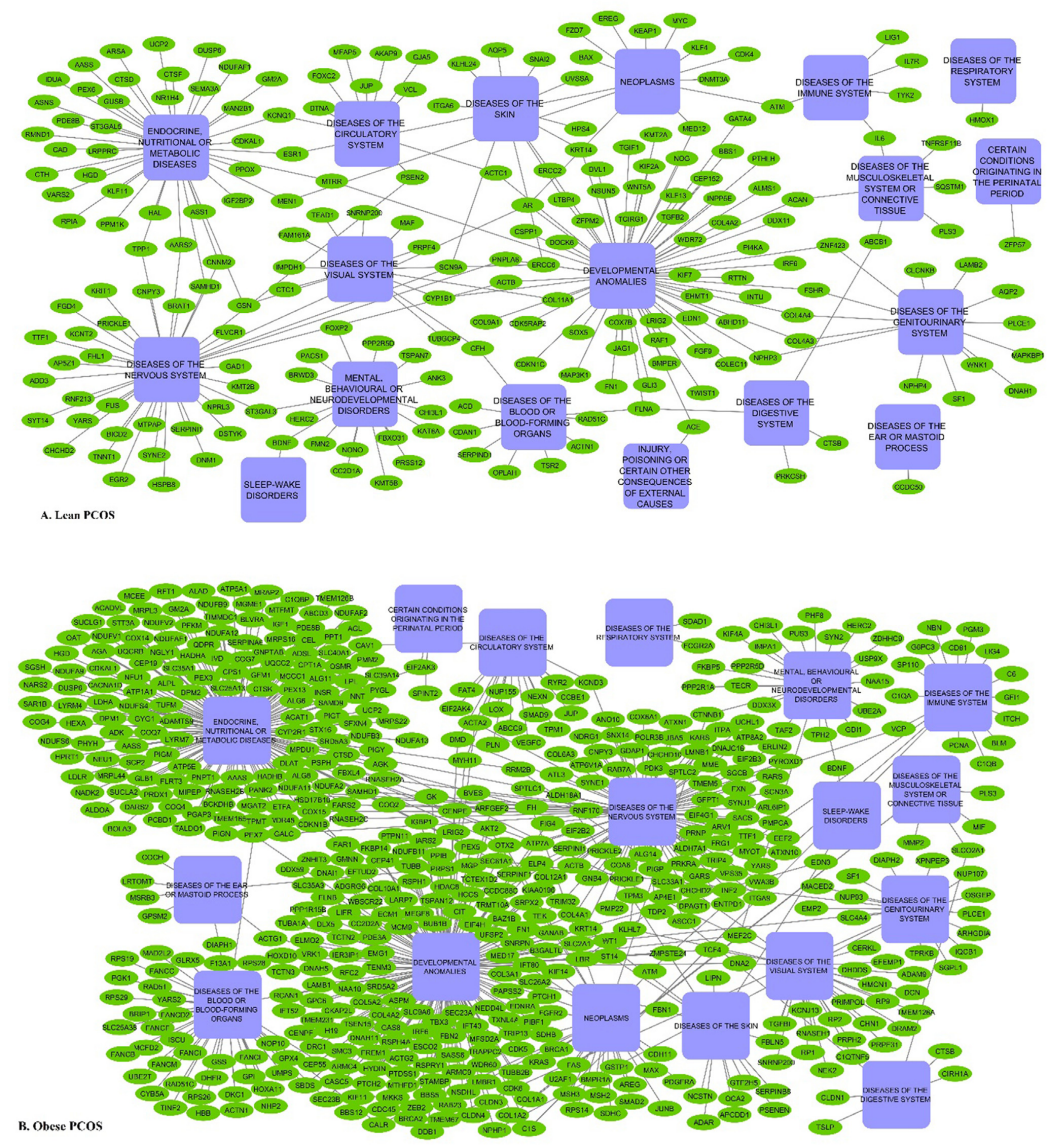
Gene-disease network for DEGs from lean (A) and obese (B) PCOS. The light-blue nodes represent the ICD-11 disease categories. Green nodes represent the DEGs. Edge represents a gene-disease association. (For interpretation of the references to colour in this figure legend, the reader is referred to the web version of this article.)

To visualize the DEGs-disease mapping as a map to support hypothesis generation, we constructed gene-disease networks for lean and obese PCOS. Although the disease categories that mapped to DEGs of lean and obese PCOS were similar, the number of DEGs associated with the diseases was much higher in obese PCOS as compared to lean PCOS (Fig. 6, Supplementary Table S9).

#### 3.5.2 Disease gene set enrichment analysis suggests divergent etiology of lean and obese PCOS

GSVA was performed to assess the association of the dysregulated genes identified in ovarian tissue samples of lean and obese PCOS (GPL570 platform only) to reproductive and endocrine diseases. The genes associated with the disease category of pregnancy, childbirth or the puerperium were upregulated in the cumulus cell and metaphase-II oocytes derived from obese PCOS as compared to non-PCOS samples. Conversely, these genes were downregulated in cumulus cell derived from lean PCOS as compared to non-PCOS samples. The difference in the gene expression pattern in cumulus cells obtained from lean women and women with obesity and PCOS suggests that the etiology of PCOS may be different in the lean and obese PCOS (Supplementary Fig. S3). The genes associated with these reproductive and endocrine diseases were traced to the GSEA pathway enrichment plots to identify the pathways associated with these genes and compare the enrichment status of these pathways across lean and obese PCOS (Supplementary Tables S10 and S11).

## 4 Discussion

PCOS has a multigenic etiology and women with PCOS are at risk of multiple comorbidities that include obesity, cardiovascular diseases and insulin-resistant diabetes.

Insulin resistance, leading to hyperinsulinemia and type-2 diabetes, is frequently observed in women with PCOS [52], [53], [54]. The altered gene expression of multiple tissues; such as ovarian, adipose and skeletal muscle; is known to contribute to this complex pathophysiology [53], [55]. Hyperinsulinemia also contributes to ovarian androgen excess, another common feature in women with PCOS [52], [54]. Insulin, along with LH, can elevate the levels of intracellular cAMP concentration in thecal cells leading to increased expression of steroidogenic genes and androgen biosynthesis [10], [54]. Insulin signaling, hyperinsulinemia and androgen synthesis are influenced by molecular pathways such as PI3K, MAPK and lipid metabolism [54], [56], [57], [58]. Nine pathways were found to be enriched in our *meta*-analysis. A list of enriched pathways, associated DEGs and literature evidence for the association of these pathways with PCOS can be seen in Supplementary Table S12. Several research groups are working on potent insulin sensitizers such as inositol and its stereoisomers [57], [59], [60], that could be effectively used in the treatment protocol for women with PCOS.

While there are numerous high throughput studies that aim to delineate the genes and pathways that are dysregulated in obese PCOS and PCOS in general, the studies on lean PCOS are scarce and studies that compare obese and lean PCOS are far scarcer [19]. Another important aspect that is not well studied is the comparative analysis of tissue-based gene dysregulation in PCOS.

In the present study, we have attempted to address the above two aspects by collating information from literature and performing a *meta*-analysis of gene expression studies and pathways for lean and obese PCOS from diverse human cell types/tissue sources and array platforms. The salient observations from the *meta*-analysis are discussed below.

1) The pathophysiology of lean and obese PCOS seems to be different

Although the majority of DEGs associated with PCOS seems to be downregulated across the lean and obese PCOS phenotypes; the expression profile of genes from similar cell types seems to be different between lean and obese PCOS (Supplementary Fig. S3). Moreover, we did not find any commonly dysregulated genes between lean and obese PCOS datasets (Supplementary Table S5). The genetic contributors of lean and obese PCOS may therefore be different. However, additional research would be required to substantiate this hypothesis. The pathway analysis also reveals that the etiology of lean and obese PCOS is different (Table 2).

2) PCOS is caused mainly by downregulation rather than upregulation of associated genes

Analysis of DEGs across tissue types for lean and obese PCOS revealed that the majority of them were downregulated (Fig. 2). GSVA analysis too indicated that the genes associated with reproductive and endocrine diseases were downregulated in both lean and obese PCOS (Supplementary Fig. S3); a trend that has been observed for most comorbid disease-associated genes. This observation is in agreement with previous studies on PCOS that have documented that most of the genes associated with PCOS were found to be downregulated in the disease state [61], [62]. Expectedly, many of these downregulated genes are enriched in relevant pathways for PCOS such as apoptosis [63], angiogenesis [64], oxidative-stress [65], glucose metabolism [66], steroid metabolism [67], immune response [68] and circadian rhythm [69].

3) Ovarian and endometrial tissues share several commonly dysregulated genes

Ovary is considered as the most important target organ of PCOS [63]. Our analysis revealed that endometrial and ovarian gene expression is considerably altered in PCOS (Fig. 2) and many of the DEGs were shared between the two sources (Fig. 3). Women with PCOS are known to suffer from infertility/subfertility which could be attributed to a) reduced oocyte/embryo quality and/or b) impaired endometrial support for embryo implantation and growth [64]. The high number of DEGs of endometrial origin obtained in our study suggests that compromised embryo implantation may be an important contributing factor for the poor reproductive outcome observed in PCOS patients*.*

4) Pathway analysis reveals that cellular homeostasis is disrupted in lean and obese PCOS

Pathway analysis revealed that several fundamental pathways responsible for cell proliferation and survival such as those involved in gene expression, DNA recombination, cell cycle, cell structure and signaling are perturbed in lean and obese PCOS (Fig. 4). A striking observation is that the nuclear-mitochondrial crosstalk seems to be differently impaired in lean and obese PCOS (Table 2). The mitochondrial biogenesis and translation machinery is heavily dependent on cues from the nucleus [65] and physical activity [66]. Previous studies have demonstrated that obesity is associated with downregulation of mitochondrial transcripts [67], lower ATP synthesis and decreased insulin sensitivity [68]. Alternatively, upregulation of mitochondrial pathways would lead to higher mitochondrial respiration and thereby increased reactive oxygen species (ROS) production [69]. Thus, both lean and obese PCOS phenotypes seem to risk the adverse effects of impaired mitochondrial translation, albeit through different manifestations.

5) The gene-disease network is denser for obese PCOS as compared to lean PCOS

Gene-disease networks mapped for dysregulated genes of obese PCOS are denser as compared to lean PCOS (Fig. 6). This data suggests that obese PCOS individuals may be at significantly higher risk of comorbidities as compared to lean PCOS.

### 4.1 Limitations of the study

An important limitation of the study is that the analysis and conclusions presented here are dependent on the gene expression data on lean and obese PCOS existing in the public domain and the consistency maintained across the datasets for phenotype annotations. The study would have benefited with higher number of tissue and BMI-matched samples than the present availability in the GEO database. A second limitation, specifically for the comorbidity analysis, is the lack of a comprehensive and updated gene-disease association databases available for researchers. For example, although the association of of *CYP1B1* in PCOS is well documented in literature, [70], [71], [72] this association is not documented in the KEGG database. Finally, this study is limited to analysis of coding genes and its association with PCOS. We have not included PCOS studies related to RNASeq, lncRNAs, drug-treated samples, cell line and non-human samples in this *meta*-analysis, which may have led to interesting observations.

## 5 Conclusions

Tissue-based comparative analysis of the DEGs, pathway networks and GDS revealed that endometrium and ovary are important target organs of PCOS. The analysis offers evidence as to why women with obesity and PCOS are at higher risk of suffering from comorbidities as compared to lean PCOS. While the unifying mechanisms of obesity, metabolic-related disorders and PCOS are still unclear [73], this study has led to identification of potential biomarkers and further research is required to establish the diagnostic and therapeutic applications of the identified pathways and gene networks for PCOS and its comorbidities.

## Data availability

The R codes used for the meta-analysis are freely available at https://github.com/bic-nirrh/pcos-metaanalysis.

## Authors contribution

S-IT was involved in study plan, data analysis and writing the manuscript; UG and SB were involved in literature review and manuscript writing; UG and KP was involved in *in silico* analysis; GDB was involved in study plan and writing the manuscript.

## CRediT authorship contribution statement

**Susan Idicula-Thomas:** Conceptualization, Methodology, Formal analysis, Writing - original draft, Writing - review & editing, Funding acquisition. **Ulka Gawde:** Methodology, Formal analysis, Writing - review & editing. **Sameeksha Bhaye:** Investigation, Writing - review & editing. **Khushal Pokar:** Formal analysis. **Gary D. Bader:** Conceptualization, Methodology, Writing - review & editing, Funding acquisition.

## Declaration of Competing Interest

The authors declare that they have no known competing financial interests or personal relationships that could have appeared to influence the work reported in this paper.
