# Supplementary material for: Meta-analysis of gene expression profiles of lean and obese PCOS to identify differentially regulated pathways and risk of comorbidities
Source: Comput Struct Biotechnol J. 2020 Jun 21;18:1735–45. doi: 10.1016/j.csbj.2020.06.023 (PMC7352056; doi:10.1016/j.csbj.2020.06.023)
Supplement: Supplementary data 1 [file mmc1.docx]

**Supplementary Figure S1: Unique pathways in lean and obese PCOS of GPL570**

1. Unique pathways in Lean PCOS (GPL570)

| 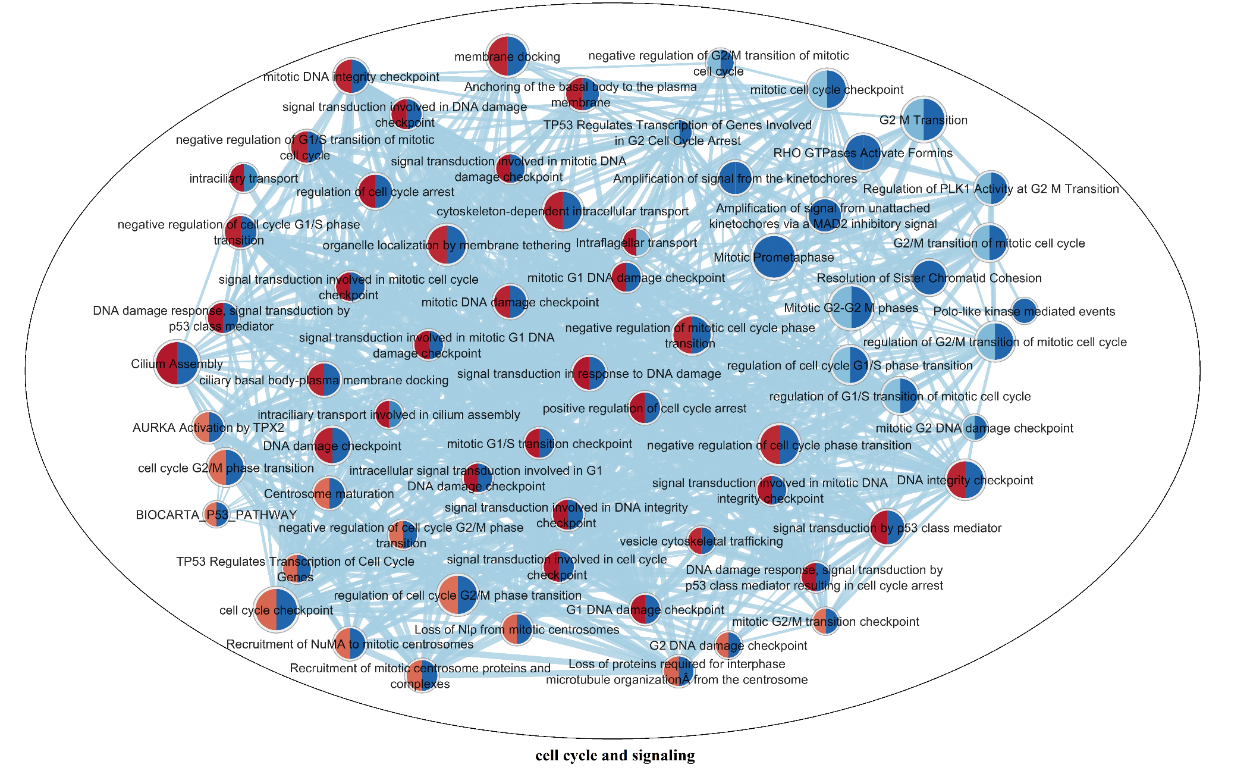  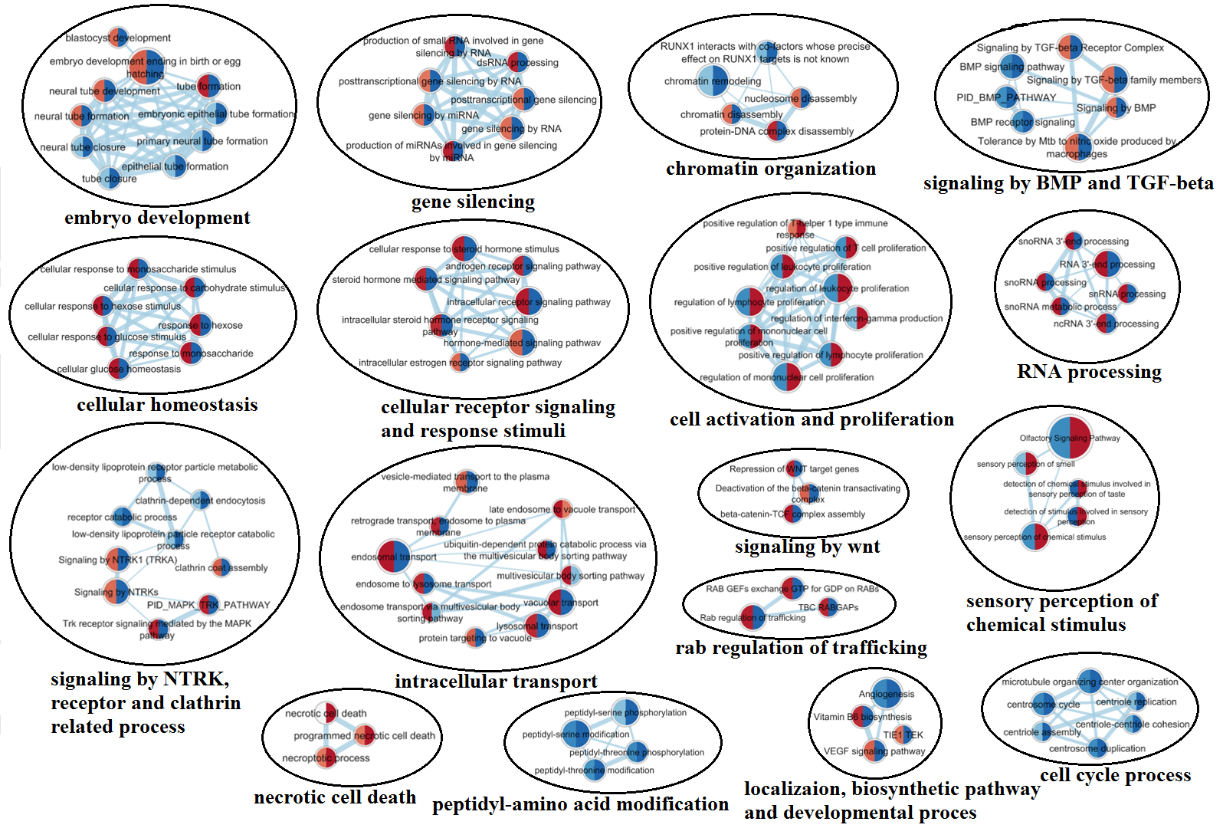  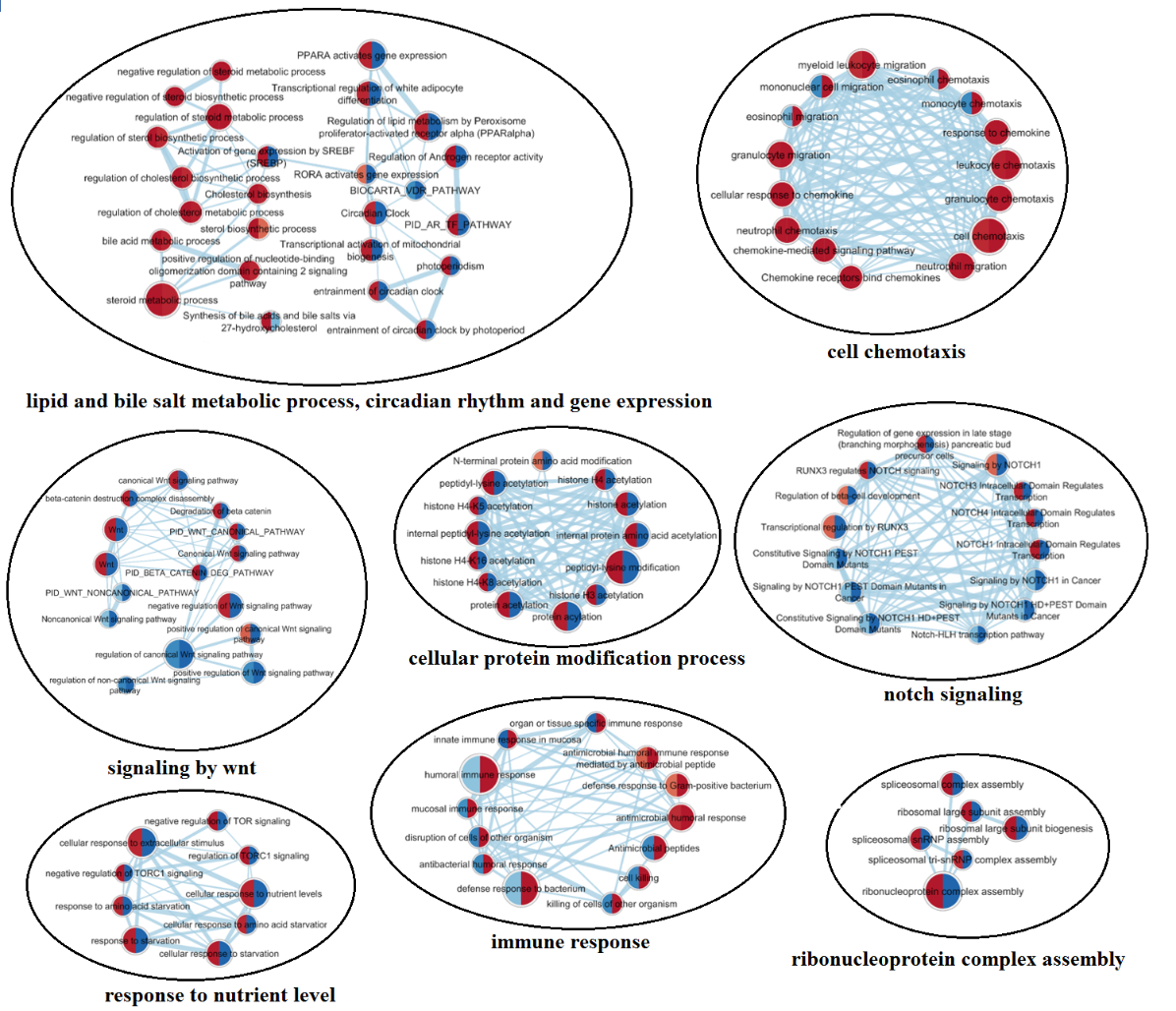  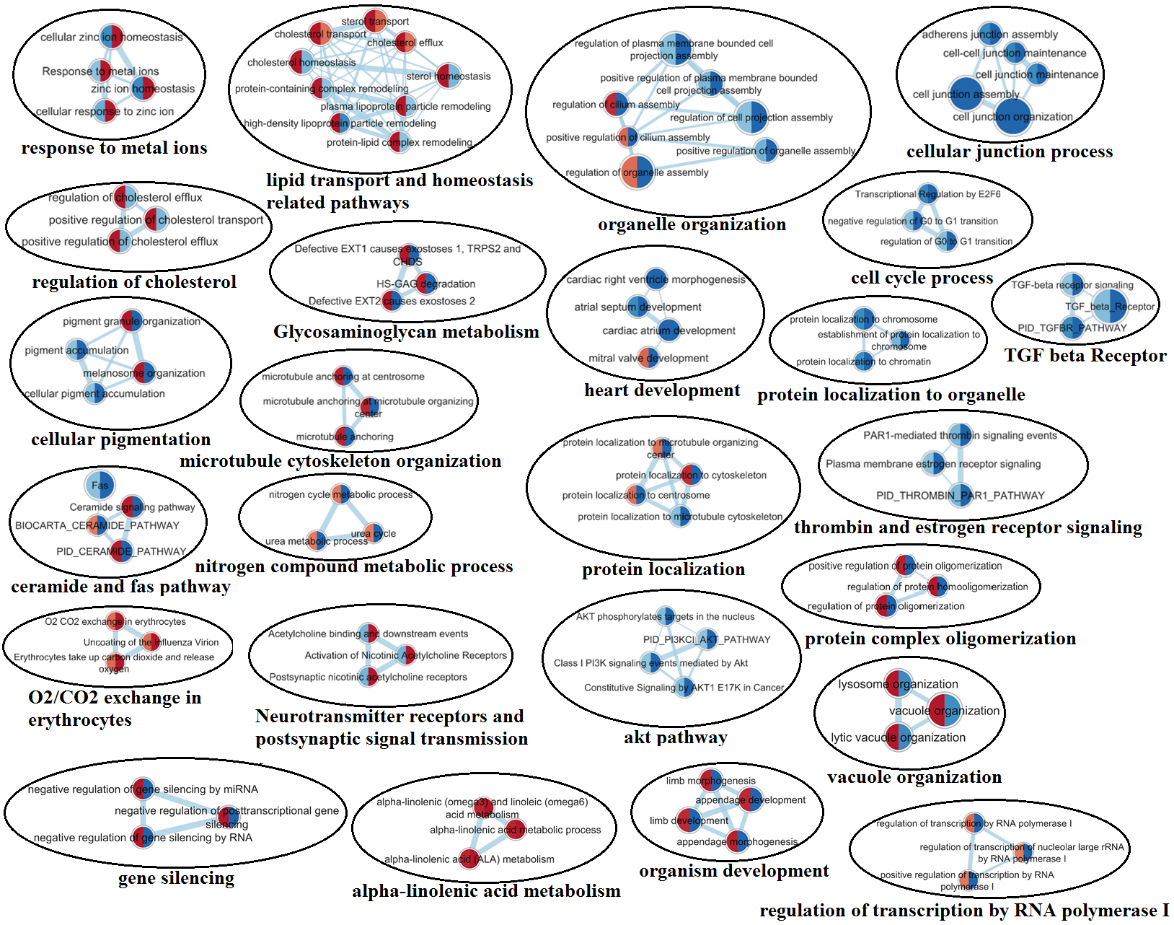  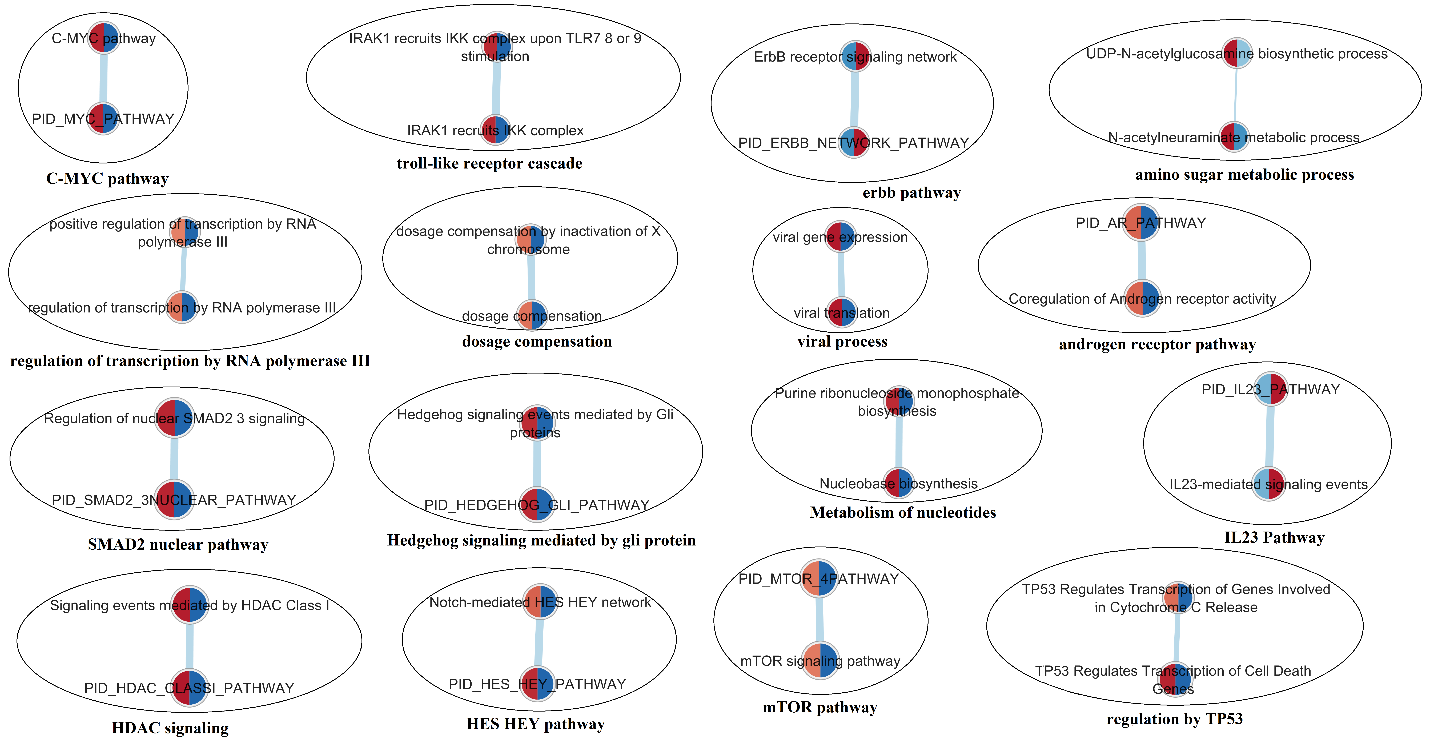  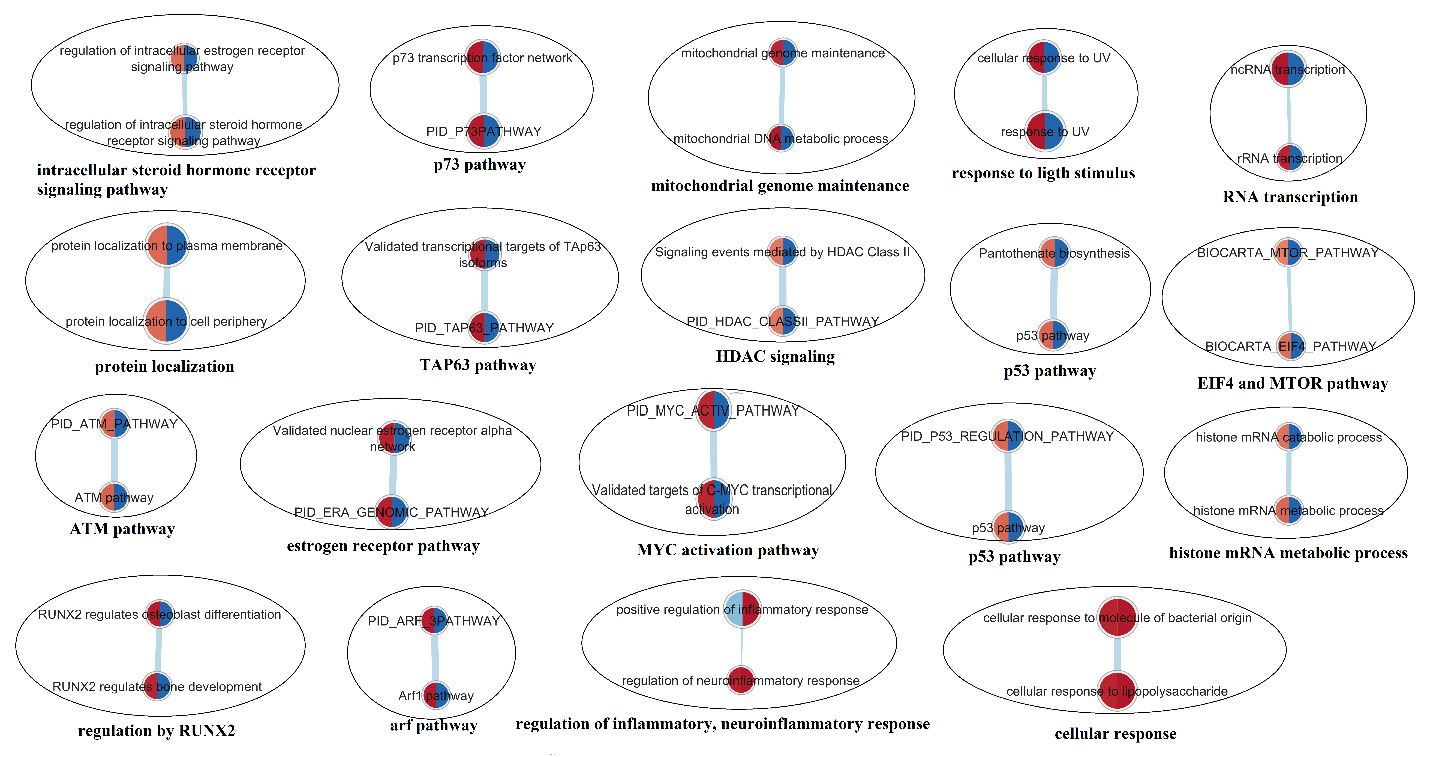  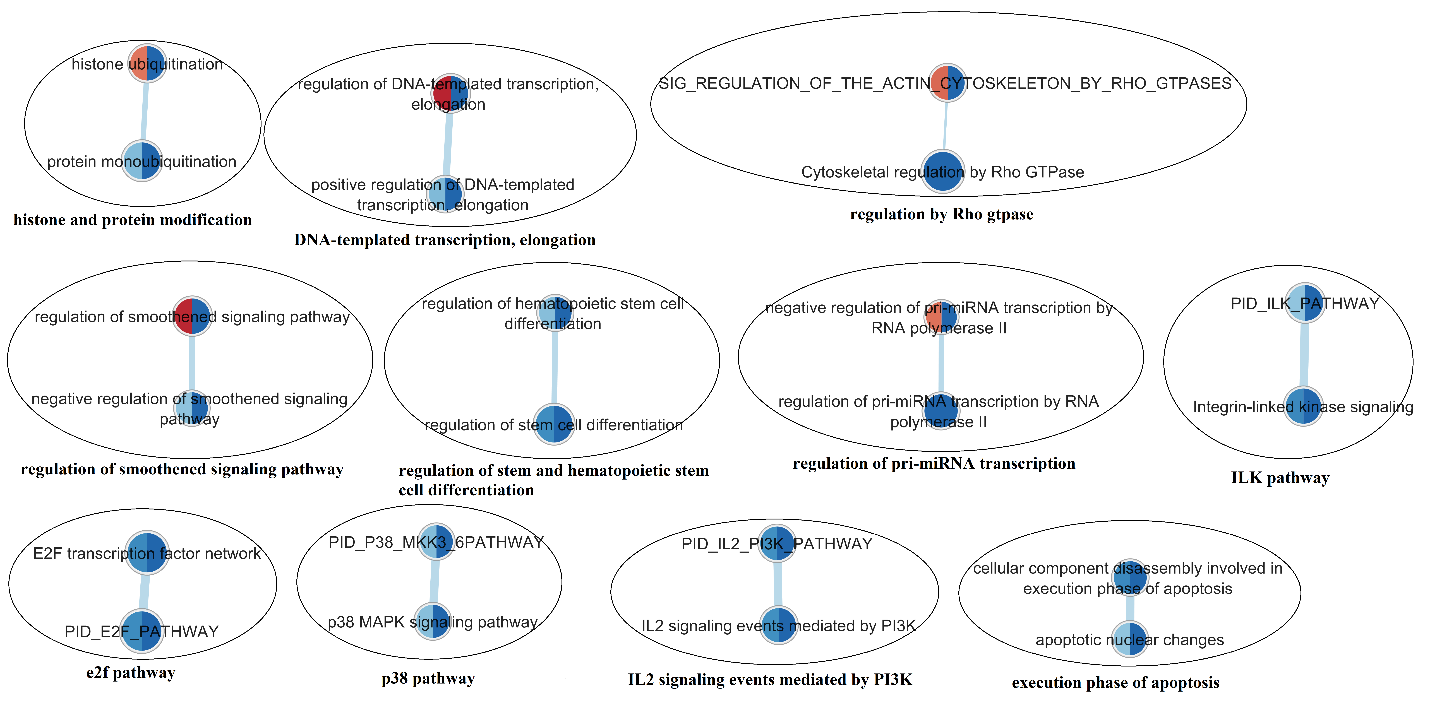  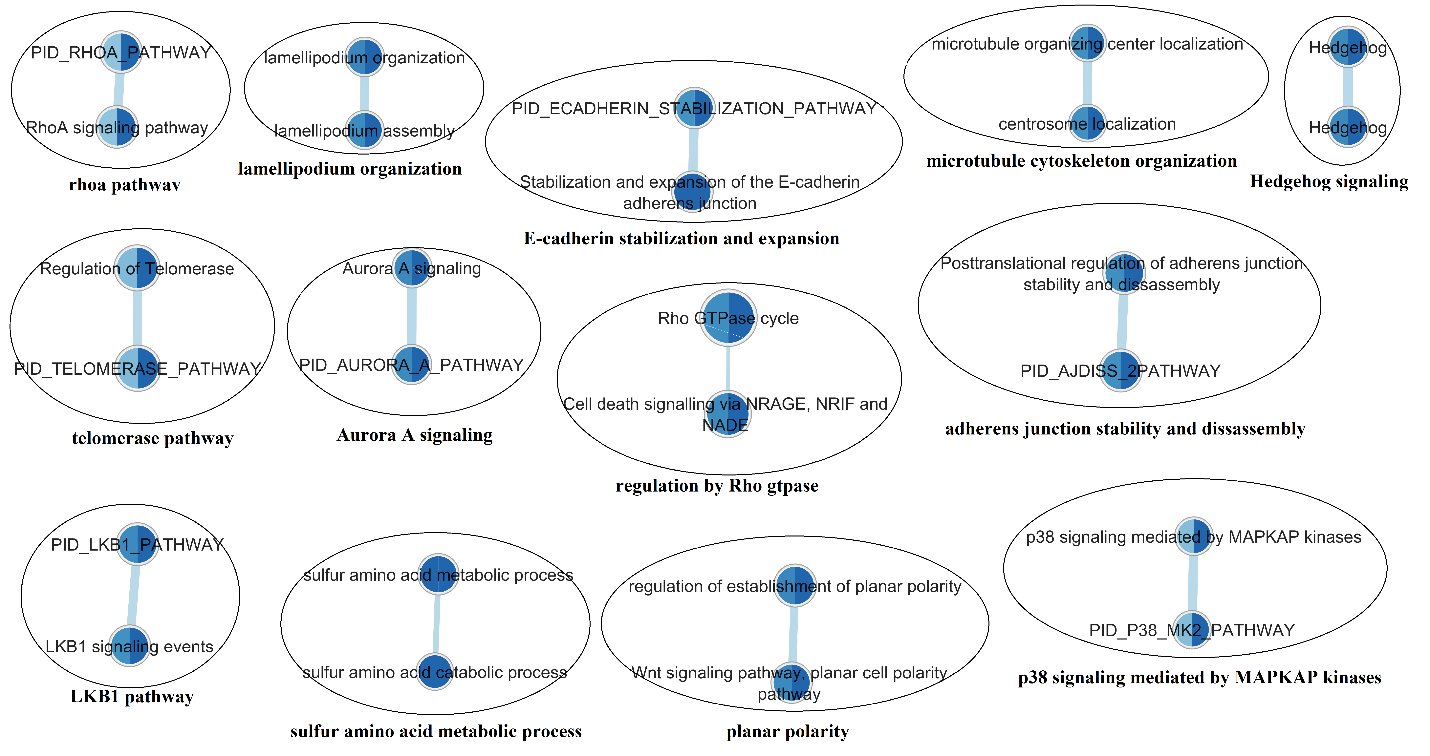 |
| --- |

1. Unique pathways in obese PCOS (GPL570)

| 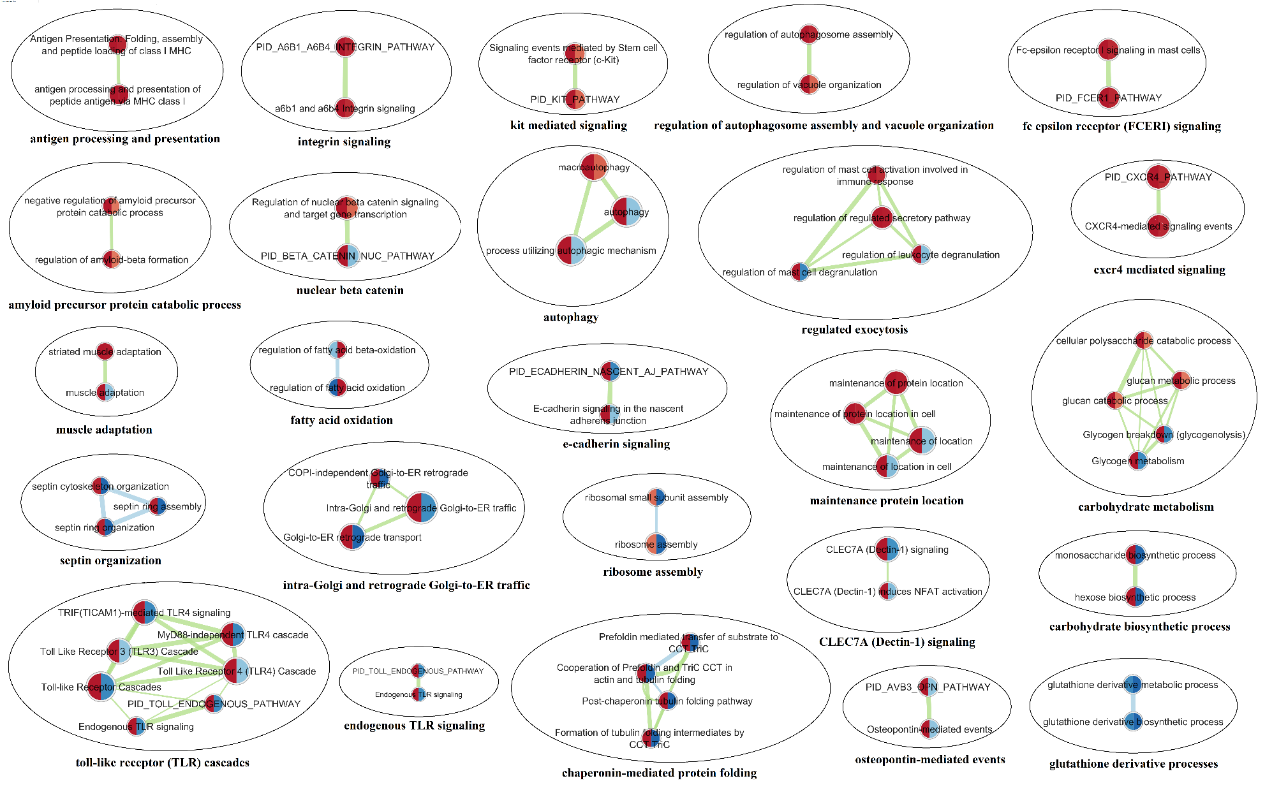 |
| --- |

**Supplementary Figure S1: Unique pathways in A) lean PCOS and B) obese PCOS of GPL570. Size of the node is indicative of the number of genes involved in the pathway. An edge between nodes represents shared genes between the pathways. Each node is segmented into two halves representing the two constituent GEO datasets (GSE10946 and GSE98421 for lean PCOS; GSE10946 and GSE6798 for obese PCOS) of GPL570 and is colored based on the NES values obtained from GSEA analysis. Red and blue represents upregulation and downregulation respectively.**
