# Supplementary material for: Meta-analysis of gene expression profiles of lean and obese PCOS to identify differentially regulated pathways and risk of comorbidities
Source: Comput Struct Biotechnol J. 2020 Jun 21;18:1735–45. doi: 10.1016/j.csbj.2020.06.023 (PMC7352056; doi:10.1016/j.csbj.2020.06.023)
Supplement: Supplementary data 2 [file mmc2.docx]

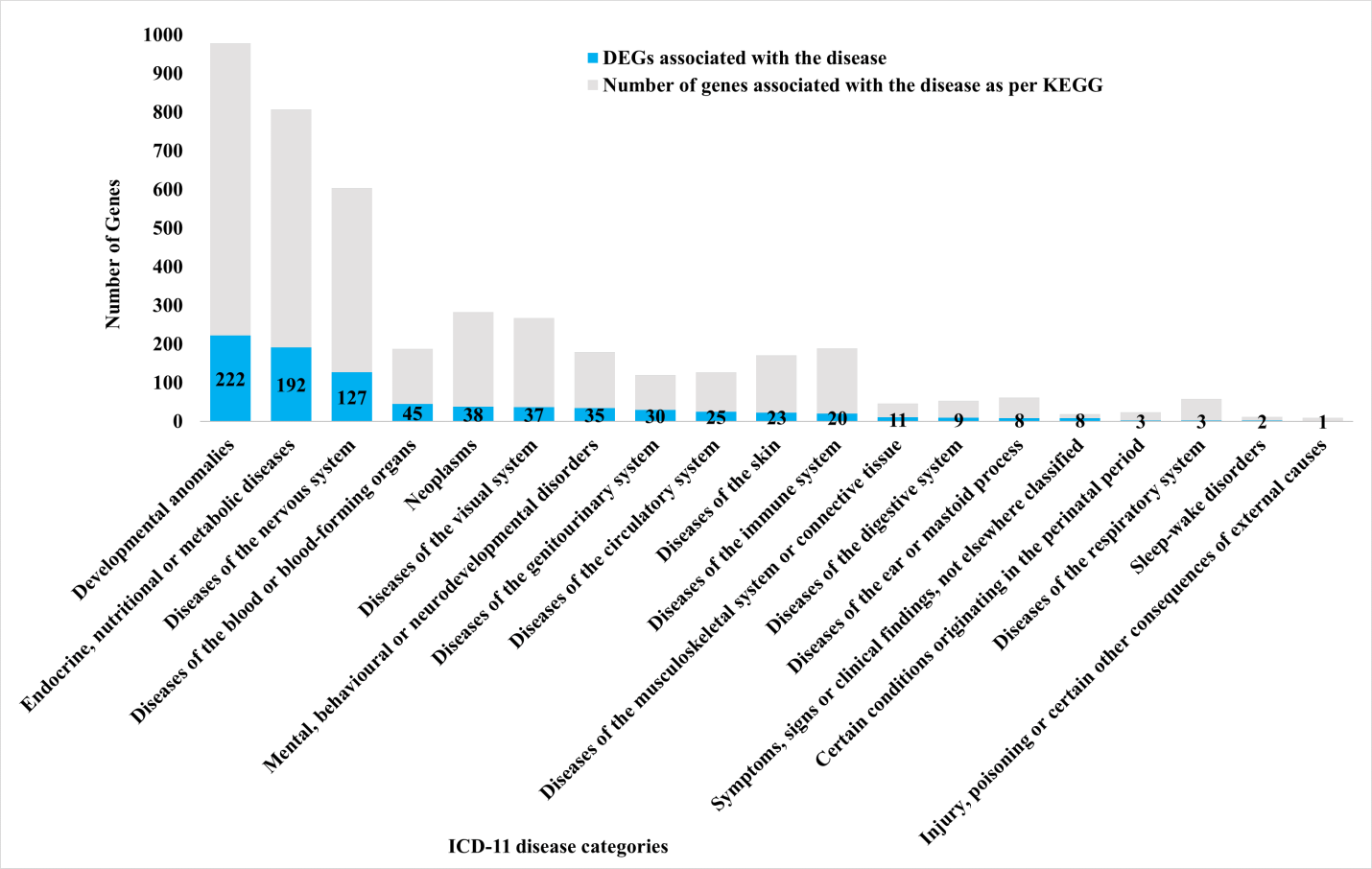


**Supplementary Figure S2. DEGs mapped to genes associated with ICD-11 disease categories. Grey bars indicate the total number of genes associated with the disorder as per the KEGG database. Blue bars indicate the DEGs mapped to the disease associated genes.**
