# Supplementary material for: Meta-analysis of gene expression profiles of lean and obese PCOS to identify differentially regulated pathways and risk of comorbidities
Source: Comput Struct Biotechnol J. 2020 Jun 21;18:1735–45. doi: 10.1016/j.csbj.2020.06.023 (PMC7352056; doi:10.1016/j.csbj.2020.06.023)
Supplement: Supplementary data 3 [file mmc3.docx]

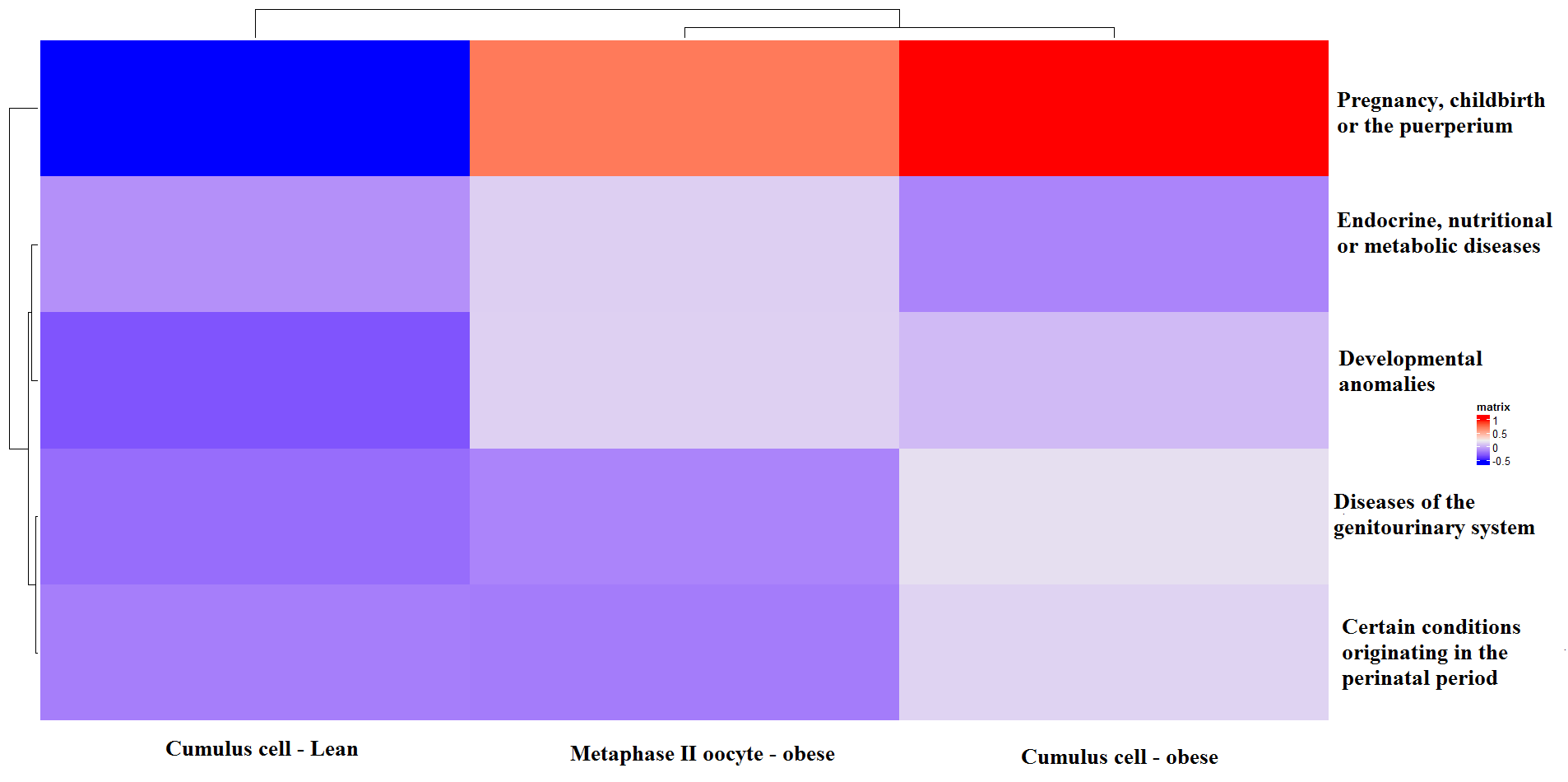


**Supplementary Figure S3. Heatmap for disease distribution across the ovarian tissue types for lean and obese PCOS (GPL570 platform only). Red represents upregulation (GSVA scores>0) and blue represents downregulation. Most of the genes associated with the diseases are seen to be downregulated for lean and obese PCOS.**
