# Supplementary material for: Meta-analysis of gene expression profiles of lean and obese PCOS to identify differentially regulated pathways and risk of comorbidities
Source: Comput Struct Biotechnol J. 2020 Jun 21;18:1735–45. doi: 10.1016/j.csbj.2020.06.023 (PMC7352056; doi:10.1016/j.csbj.2020.06.023)
Supplement: Supplementary data 7 [file mmc7.docx]

**Supplementary Table S4: Transcription factor analysis of DEGs using iRegulon**

| **Motif** | **NES** | **Transcription Factor** | **Tissue source** | **Regulation in Lean PCOS** | **Regulation in obese PCOS** |
| --- | --- | --- | --- | --- | --- |
| transfac_pro-M01983 | 3.28 | *ETV3* | Endometrial epithelial (GSE48301) obese | Absent | Downregulated |
|  |  | *GABPB1* | Endometrial mesenchymal (GSE48301) obese | Absent | Downregulated |
|  |  | *ELF3* | Endometrial epithelial (GSE48301) obese | Absent | Downregulated |
|  |  | *GABPA* | Lutein granulosa cells (GSE98595) lean | Downregulated | Absent |
|  |  | *ELF1* | Cumulus Cell (GSE10946) lean | Downregulated | Absent |
|  |  | *ELF4* | Cumulus Cell (GSE10946) lean | Downregulated | Absent |
| transfac_pro-M01007 | 3.27 | *SRF* | Cumulus Cell (GSE10946) lean | Downregulated | Absent |
