# Supplementary material for: Meta-analysis of gene expression profiles of lean and obese PCOS to identify differentially regulated pathways and risk of comorbidities
Source: Comput Struct Biotechnol J. 2020 Jun 21;18:1735–45. doi: 10.1016/j.csbj.2020.06.023 (PMC7352056; doi:10.1016/j.csbj.2020.06.023)
Supplement: Supplementary data 8 [file mmc8.docx]

**Supplementary Table S5: Commonly dysregulated genes in PCOS across sample types and GEO platforms**

| **Phenotype** | **Platform** | **DEGs** | **Regulation Status** |
| --- | --- | --- | --- |
| **Lean** | All data combined (GPL570, GPL6244) | *Nil* | - |
|  | GPL570 only | *PRRT1* | Downregulated (cumulus cells and subcutaneous adipose) |
|  |  | *SLITRK4* | Downregulated (cumulus cells and subcutaneous adipose) |
|  |  | *CRHBP* | Downregulated (cumulus cells and subcutaneous adipose) |
|  |  | *HAPLN1* | Upregulated (cumulus cells); Downregulated (subcutaneous adipose) |
|  |  | *SRGN* | Upregulated (cumulus cells); Downregulated (subcutaneous adipose) |
|  |  | *EREG* | Upregulated (cumulus cells); Downregulated (subcutaneous adipose) |
|  |  | *WNT5A* | Upregulated (cumulus cells); Downregulated (subcutaneous adipose) |
| **Obese** | All data combined (GPL570, GPL6244, GPL96, GPL15362) | *Nil* | - |
|  | GPL570 only | *Nil* | - |
