# Supplementary material for: Meta-analysis of gene expression profiles of lean and obese PCOS to identify differentially regulated pathways and risk of comorbidities
Source: Comput Struct Biotechnol J. 2020 Jun 21;18:1735–45. doi: 10.1016/j.csbj.2020.06.023 (PMC7352056; doi:10.1016/j.csbj.2020.06.023)
Supplement: Supplementary data 9 [file mmc9.docx]

**Supplementary Table S6: DEGs obtained from tissue-based analysis**

| **Tissue source** | **No. of common DEGs** | ***DEGs*** |
| --- | --- | --- |
| Ovarian, endometrial and adipose tissues | 6 | *SRGN, TMEM256, GPX7, MAT2A, SVIP, SERPINI1* |
| Ovarian and endometrial tissues | 181 | *SLC7A2, POLR1E, CHURC1, KATNBL1, CDKAL1, PDE8B, POTEKP, EIF4A1, JUP, ZMYM1, AASS, SLC44A2, PARD6G, KIF27, GTF2H3, CLASP2, TTF1, ATM, PAMR1, GUSBP3, PRKAB2, PRICKLE1, VPS8, SMPD4, ST6GALNAC3, FN1, SNRNP200, CHCHD2, DAB2, LCMT1, SET, TRMT2B, ARPC3, ACTB, UTP20, ACTN4, PLS3, DCLRE1A, PMM1, CNPY3, PUM1, UCP2, TMEM259, PHACTR4, ANXA4, SIMC1, SF1, MORN4, NUP133, ZNF561, TSPAN5, KLHDC3, IRF6, CPXM1, AES, QRSL1, COL4A2, CAND1, ARHGAP35, ALDH1A2, PRR11, AMY2B, ACTN1, TBCEL, CTSD, SCG5, NQO2, LRIG2, BTBD6, LINC01128, GSR, RALGPS2, LAYN, HSPA1B, YARS, CRYZL1, HERC2, WLS, RRAGC, HINT3, PPP2R5D, WDR59, SFRP1, SDHAF2, MTFMT, MRPL57, FKBP5, GALC, L3HYPDH, SLC44A1, NDUFA11, PTDSS1, RNF145, WDR41, DYRK4, UQCR10, TMEM242, RANBP1, SNRNP40, PLBD1, BLM, FAU, APOO, MAPK14, CPT1A, MRPL22, BVES, MED27, MRPS23, POLE4, CDCA2, PKNOX1, ARHGAP18, NUTF2, ITGB1BP1, SHCBP1, SF3B5, CADPS2, BMPR1A, CEP70, CBLN4, PDIA6, ARL6IP1, ZNF814, LRRTM4, CLDND1, SETDB2, TWISTNB, NEDD1, COQ2, NSMCE4A, U2AF1, AGA, SCAMP2, DDX59, NUMA1, IGFBP3, RSPRY1, PSD3, WBP4, APH1A, MSH3, DCK, DPM1, CD99, TMEM231, PBK, DPH6, CAPZB, UBE2D1, DDB1, WDR45B, DET1, FAM107B, SPRY2, ZFP36, DNAJC15, C2orf43, RNA5SP405, CD24, SNORD49A, C3orf14, FAM216B, IFI27, NDUFAF1, DEXI, NR4A1, MICU2, DUSP6, NDUFB6, LRIG3, AS3MT, GM2A, TCEB1P2, PDCL3, SNRPG, SAMHD1, MRPL15, BTG2, RPL22L1, RNA5SP192* |
| Ovarian and adipose tissues | 21 | *HAPLN1, EREG, WNT5A, CRHBP, NEAT1, SLITRK4, PRRT1, ZNF541, OAZ3, POPDC3, SNORD65, DAPL1, SHOX2, MRPL41, KIAA1671, UGCG, SMIM37, ZNF581, LINC00467, CRISPLD2, THNSL2* |
| Endometrial and adipose tissues | 45 | *MYH10, SLC6A6, IMMP1L, S100A2, MRPS33, ACTA2, PCDHB3, CTSB, KCTD20, NLGN1, COPA, ULK4, IGFBP7, PLCE1, SCIN, HGD, DIAPH3, UQCRB, CHSY3, EDIL3, TMTC1, ACTG2, EPHA5, APOLD1, LRRC17, NDUFAF2, COX7A2P2, HHIP, ZCCHC10, TCF21, BDNF, NDUFB11, FHL2, UQCRH, MEST, NFU1, MRPS36, COX7A1, PTRHD1, THBS2, MRPL52, PPP1R14A, TOX, OSMR, PIGP* |
| Endometrial and skeletal tissues | 5 | *SLC38A1, ALDH6A1, H19, C12ORF75, C9ORF135* |
