# Supplementary material for: Meta-analysis of gene expression profiles of lean and obese PCOS to identify differentially regulated pathways and risk of comorbidities
Source: Comput Struct Biotechnol J. 2020 Jun 21;18:1735–45. doi: 10.1016/j.csbj.2020.06.023 (PMC7352056; doi:10.1016/j.csbj.2020.06.023)
Supplement: Supplementary data 11 [file mmc11.docx]

**Supplementary Table S8: DEGs associated with ICD-11 disease categories**

| **Disease categories** | **No. of DEGs** | **DEGs** |
| --- | --- | --- |
| Developmental anomalies | 222 | *TCTN3, ACTC1, CEP55, JAG1, PIBF1, B3GALTL, TSPAN12, TCIRG1, MED12, IFT80, SOX5, ERCC6, EHMT1, CKAP2L, LARP7, BUB1B, ACAN, CSPP1, ERCC2, PEX7, WNT5A, GMNN, FBN2, NOG, ZFPM2, CCDC88C, EDN1, CDK5RAP2, KIAA0196, CYP1B1, GPC6, ACTB, KRT14, FIG4, AR, SASS6, CIT, TGFB2, KIF11, GLI3, FLNB, HCCS, RAB23, ZNHIT3, COL4A2, CEP152, ESCO2, DOCK6, CENPE, COL4A3, ASPM, IRF6, UFSP2, MKKS, INPP5E, WDR72, MAP3K1, COLEC11, KIF14, SNRPN, KIF2A, NPHP3, TGIF1, TWIST1, FGF9, GATA4, TCTEX1D2, ACE, MTRR, COL11A1, TXNL4A, RTTN, CASC5, FSHR, DNA2, ZNF423, PI4KA, IGBP1, TRIP13, BBS1, INTU, TSEN15, CENPF, LRIG2, KLF13, BMPER, FN1, NSUN5, PNPLA6, WDR60, FAR1, EFTUD2, GK, ELMO2, ST14, DNAH11, FLNA, EDN3, SRPX2, SEC23A, CDK5, RAF1, PPP1R15B, EIF4H, CLDN3, KLHL7, PTCH2, COL1A2, TUBB, PRPS1, GAS8, ZMPSTE24, TRAPPC2, MFSD2A, PEX5, SLC9A6, TMEM67, NPHP1, TCF4, RSPH4A, LBR, IARS2, NEDD4L, WBSCR22, HOXD10, PPIB, ECM1, SRD5A2, ZEB2, CEP41, C1S, IFT43, CLDN4, WT1, MEF2C, ARMC9, PTCH1, DNAI1, RSPH1, BBS5, COL1A1, ACTG1, GANAB, COL5A2, TUBA1A, CDK6, TEK, SEC23B, MGP, KMT2A, SERPINF1, COL3A1, FBN1, CC2D2A, DRC1, DDX11, EDNRA, AKT2, COL9A1, DDX59, SMC3, RSPRY1, BAZ1B, SDHB, ELP4, COL12A1, LIFR, ATP7A, TMEM231, MYH11, ACTG2, TENM3, MTHFD1, ACTA2, STAMBP, DDB1, ABHD11, COX7B, PTHLH, H19, PDE3A, TRMT10A, LTBP4, MCM9, CDC45, COL4A4, ALMS1, PAPSS2, TBX3, ADGRG6, ARFGEF2, OTX2, RCAN1, IFT52, KRAS, FREM1, IER3IP1, MEGF8, SLC35A3, SLC26A2, LAMB1, NSDHL, ARMC4, DVL1, VRK1, COL4A1, MED17, HYDIN, TRIM32, TCTN2, NDUFB11, HDAC8, TUBB2B, EMG1, FKBP14, RFC2, KIF7, PTDSS1, NAA10, GPX4, CDKN1C, DNAH5* |
| Endocrine, nutritional or metabolic diseases | 192 | *NDUFA2, ABCD3, MAN2B1, RNASEH2C, NDUFAF2, NEU1, UQCRB, FARS2, PCBD1, PPOX, ALG11, COX15, COG4, HEXA, MRPS22, PEX3, PIGT, SEC61A1, IGF2BP2, STT3A, SUCLA2, SLC25A13, PNPT1, PANK2, BCKDHB, MPDU1, GSN, HADHB, NDUFA9, IDUA, COQ2, SCP2, AASS, CYP2R1, CAD, MRPL3, LPL, MGME1, CTH, SAMHD1, FBXL4, CEP19, CPS1, GM2A, TPMT, NR1H4, DUSP6, NFU1, OSMR, CPT1A, RMND1, ATP7A, NNT, DPM1, KLF11, AGL, SFXN4, AGA, AKT2, ALG8, NADK2, OTX2, PRDX1, TIMMDC1, TUFM, CACNA1D, MIPEP, RPIA, PIGM, MRPS16, EIF2AK3, GK, PMM2, LDHA, FAR1, AAAS, ALPL, ACADVL, HADHA, DPM2, CDKN1B, CEL, SERPINA6, SLC39A14, COG7, CNNM2, WDR45, COQ4, GLB1, GNPTAB, ALDH18A1, GALC, ATP1A1, CTSK, LDLR, PIGN, NDUFV1, IGF1, STX16, ATP5A1, ESR1, NDUFA11, OAT, PGAP3, MCCC1, PEX13, TMEM165, PFKM, MTFMT, DARS2, NDUFA13, RFT1, RRM2B, SGSH, ALDOA, GFM1, ADAMTS9, PPT1, PEX5, AGK, INSR, TALDO1, SLC40A1, HPRT1, RNASEH2B, MRAP2, ATP5E, SEMA3A, BOLA3, NDUFS6, NDUFA12, NDUFB9, SAMD9, MTRR, COX14, ACAT1, C1QBP, ADK, ALG6, MGAT2, UCP2, AARS2, TMEM126B, PEX7, UQCC2, ST3GAL5, PYGL, HGD, SLC35A1, BLVRA, CDKAL1, NDUFV2, LYRM4, DLAT, FLRT3, NDUFB3, PDE8B, PIGY, PHYH, KCNQ1, NGLY1, ARSA, CTSD, ADSL, VARS2, SRD5A3, TPP1, CTSF, LYRM7, MEN1, LRPPRC, FH, GUSB, SUCLG1, ETFA, NDUFS4, PEX6, SAR1B, MCEE, COQ7, PPM1K, GYG1, HSD17B10, RNASEH2A, NDUFAF1, ASS1, ASNS, QDPR, NARS2, HAL, CAV1, MRPL44* |
| Diseases of the nervous system | 127 | *KMT2B, CNNM2, COL6A3, SPTLC2, SYNJ1, INF2, ITPA, SRPX2, RNF213, RAB7A, AARS2, PRNP, ALDH18A1, SLC2A1, KLHL7, GALC, TPP1, EIF4G1, COX6A1, RNASEH2A, ENTPD1, BICD2, DNM1, CNPY3, TNNT1, AR, KARS, GARS, GDAP1, FXN, UCHL1, TRIP4, ATL3, SNX14, MME, LMNB1, RNASEH2B, PMP22, MTPAP, SACS, ARV1, EEF2, SGCB, PRICKLE2, EIF2B2, ACTB, CHCHD2, TTF1, GAD1, AP5Z1, FRG1, ANO10, PMPCA, MYOT, PDK3, ACTA2, AP4E1, PYROXD1, TDP2, VPS35, TMEM5, COX15, GNB4, KCNT2, CHCHD10, FGD4, ATP8A2, FARS2, RNASEH2C, PIGP, COQ2, ATXN10, SERPINI1, EIF2B3, ASCC1, DNAJC16, PSEN2, PANK2, ARL6IP1, FIG4, FHL1, PRICKLE1, UBA5, RNF170, SCN3A, COL12A1, ELP4, SCN9A, ATP7A, SLC33A1, KIAA0196, CCDC88C, POLR3B, VCP, DSTYK, PNPLA6, HSPB8, ALG14, COL4A1, SYNE1, PRKRA, SYT14, BRAT1, TRIM32, FLVCR1, VWA3B, ALDH7A1, EGR2, SAMHD1, FUS, KRIT1, PRPS1, ATP6V1A, BVES, COA6, ADD3, ST3GAL3, ERLIN2, NDRG1, DPAGT1, RARS, GFPT1, SYNE2, ATXN1, YARS, ITGA9, NPRL3* |
| Diseases of the blood or blood-forming organs | 45 | *RPS28, SERPIND1, RAD51, TSR2, RAD51C, FANCB, CALR, CFH, SLC2A1, MCFD2, HOXA11, ACTN1, GPI, GSS, NHP2, BRCA2, BRIP1, CDAN1, FANCD2, UMPS, UBE2T, ISCU, NOP10, DIAPH1, TINF2, PGK1, FANCI, FANCM, FANCL, GLRX5, FANCC, DKC1, PANK2, SEC23B, YARS2, HBB, RPS19, CYB5A, FANCF, DHFR, F13A1, RPS29, FLNA, OPLAH, ACD* |
| Neoplasms | 38 | *MSH3, KEAP1, KRAS, CTNNB1, SMAD2, ESR1, SDHC, CDH11, PDGFRA, PTCH1, CDKN1B, ATM, GSTP1, AR, MED12, MEN1, CDK4, DNMT3A, BAX, FZD7, KLF4, BMPR1A, OTX2, AKT2, U2AF1, RPS14, SDHB, BRCA1, AREG, FH, KIF14, MSH2, FAS, BRCA2, EREG, MYC, JUNB, CALR* |
| Diseases of the visual system | 37 | *FBLN5, MAF, CTC1, EFEMP1, DRAM2, TGFBI, CFH, CYP1B1, DCN, FAM161A, TMEM126A, PRPF4, HMCN1, PRIMPOL, DHDDS, AGK, CHN1, PRPF31, TCF4, COL11A1, RP2, CERKL, TEAD1, RRM2B, SNRNP200, TUBGCP4, RP1, RP9, IMPDH1, KLHL7, C1QTNF5, NEK2, GSN, ADAM9, KCNJ13, DNA2, ERCC6* |
| Mental, behavioural or neurodevelopmental disorders | 35 | *PPP2R1A, ANK3, BDNF, KIF4A, TPH2, KAT6A, CC2D1A, BRWD3, PRSS12, KMT5B, PHF8, NONO, USP9X, PUS3, PPP2R5D, PACS1, UBE2A, IMPA1, ZDHHC9, ST3GAL3, FBXO31, HERC2, CHI3L1, CTNNB1, MEF2C, DDX3X, FMN2, SYN2, NAA15, GDI1, FKBP5, TSPAN7, FOXP2, TAF2, TECR* |
| Diseases of the genitourinary system | 30 | *TMEM67, NPHP1, WNK1, DIAPH2, FSHR, ZNF423, SLC4A4, ARHGDIA, EMP2, LAMB2, AQP2, NPHP4, COL4A3, CLCNKB, NUP93, SEC61A1, NPHP3, TPRKB, PLCE1, MAGED2, WT1, MAPKBP1, OSGEP, SF1, COL4A4, XPNPEP3, NUP107, IQCB1, SGPL1, DNAH1* |
| Diseases of the circulatory system | 25 | *ABCC9, CCBE1, NEXN, VEGFC, FAT4, EIF2AK4, NUP155, RYR2, LOX, JUP, CAV1, PSEN2, AKAP9, MFAP5, VCL, PLN, ACTA2, MYH11, GJA5, KCNQ1, ACTC1, TPM1, FOXC2, DTNA, SMAD9* |
| Diseases of the skin | 23 | *LTBP4, ALDH18A1, KLHL24, AQP5, EDN3, LIPN, ST14, SNAI2, UVSSA, ADAR, GTF2H5, KRT14, SCN9A, OCA2, ITGA6, ERCC6, ZMPSTE24, HPS4, NCSTN, APCDD1, FBLN5, FBN1, ERCC2* |
| Diseases of the immune system | 20 | *G6PC3, TYK2, LIG4, PGM3, BLM, IL6, NBN, C1QB, SP110, LIG1, PCNA, IL7R, ITCH, C1QA, LBR, C6, CD81, ATM, C1S, VCP* |
| Diseases of the musculoskeletal system or connective tissue | 11 | *ABCB1, COL1A1, IL6, TNFRSF11B, SQSTM1, ACAN, MMP2, MIF, SLCO2A1, PLS3, COL1A2* |
| Diseases of the digestive system | 9 | *CIRH1A, SLCO2A1, FLNA, PRKCSH, ASCC1, CTSB, TSLP, CLDN1, ABCB1* |
| Diseases of the ear or mastoid process | 8 | *MSRB3, GPSM2, ACTG1, LRTOMT, PRPS1, COCH, DIAPH1, CCDC50* |
| Symptoms, signs or clinical findings, not elsewhere classified | 8 | *FN1, ACTN4, TRPC6, INF2, ANLN, TSPYL1, CFH, APOL1* |
| Certain conditions originating in the perinatal period | 3 | *ZFP57, EIF2AK3, SPINT2* |
| Diseases of the respiratory system | 3 | *FCGR2A, SDAD1, HMOX1* |
| Sleep-wake disorders | 2 | *BDNF, EDN3* |
| Injury, poisoning or certain other consequences of external causes | 1 | *ACE* |
