# Supplementary material for: Meta-analysis of gene expression profiles of lean and obese PCOS to identify differentially regulated pathways and risk of comorbidities
Source: Comput Struct Biotechnol J. 2020 Jun 21;18:1735–45. doi: 10.1016/j.csbj.2020.06.023 (PMC7352056; doi:10.1016/j.csbj.2020.06.023)
Supplement: Supplementary data 13 [file mmc13.docx]

**Supplementary Table S10: Number of enriched pathways in ovarian tissues of GPL570 which are associated with reproductive and endocrine diseases**

| **Disease** | **Number of associated genes** | **GSE10946-Cumulus Cell - lean** | | **GSE5850-Metaphse II oocyte - obese** | | **GSE10946-Cumulus Cell - obese** | |
| --- | --- | --- | --- | --- | --- | --- | --- |
|  |  | **Upregulated** | **Downregulated** | **Upregulated** | **Downregulated** | **Upregulated** | **Downregulated** |
| Pregnancy, childbirth or the puerperium | 2 | 0 | 4 | 0 | 0 | 0 | 0 |
| Endocrine, nutritional or metabolic diseases | 750 | 71 | 589 | 10 | 0 | 7 | 21 |
| Developmental anomalies | 910 | 95 | 832 | 14 | 0 | 7 | 42 |
| Diseases of the genitourinary system | 180 | 22 | 288 | 2 | 0 | 1 | 6 |
| Certain conditions originating in perinatal period | 20 | 7 | 98 | 0 | 0 | 0 | 0 |
