# Supplementary material for: Meta-analysis of gene expression profiles of lean and obese PCOS to identify differentially regulated pathways and risk of comorbidities
Source: Comput Struct Biotechnol J. 2020 Jun 21;18:1735–45. doi: 10.1016/j.csbj.2020.06.023 (PMC7352056; doi:10.1016/j.csbj.2020.06.023)
Supplement: Supplementary data 15 [file mmc15.docx]

**Supplementary Table S12: Enriched pathways for downregulated genes present in insulin resistance and hyperandrogenism.**

| **Pathway Name** | **Pathways enriched in analysis** | **DEGs** | **Reference** |
| --- | --- | --- | --- |
| **a) Insulin resistance** | | | |
| phosphatidylinositol 3 kinase/protein kinase B (PI3K/PKB, also known as Akt) pathway | PI3KCI/ AKT pathway | *RAF1, FOXO1, TBC1D4, MAP3K5, AKT2, BCL2L1, CDKN1A, CDKN1B, PRKDC* | DOI: 10.5772/intechopen.89246 |
| mitogen-activated protein kinase/extracellular signal-regulated kinase (MAPK/ERK) pathway. | p38 MAPK pathway | *MAP3K4, ATM, GADD45G, MAP3K1, SRF, MKNK2, MAP3K5, GADD45B, MAPK14, CDC42, MAPK1, MEF2C* | DOI: 10.5772/intechopen.89246 |
|  | MAPK pathways | *MAP3K4, MYC, RAF1, MAP3K12, MAP2K5, MAP3K1, MKNK2, TGFB2, MAP3K5, JUN, FOS, MAX, MAPK14, PAK2, MAPK1, MAPK8, MEF2C, MAPK9* |  |
| mTOR signalling pathway | mTOR signalling pathway | *PPARGC1A, EIF4A1, RAF1, RRAGC, POLDIP3, EIF4EBP1, RHEB, KRAS, RRAGA, MAPK1, EIF4G1, ATG13, EEF2, BNIP3* | PMID: 29187279 |
| **b) Hyperandrogenism** | | | |
| Steroidogenic pathway | Steroid metabolic process | *CYP2E1, CYP2B6, OSBPL7, ACACB, LGMN, OSBPL5, BMP6, CYP1B1, FGF1, SNAI2, IL1B, NR1H4, GFI1, AKR1C1, FGFR4, OSBPL9, PRKAA1, OSBPL1A, TSPO, MBTPS1, PANK2, LBR, HMGCR, CYP7A1, STARD3NL, SQLE, ACLY, SRD5A2, ACADVL, NSDHL, SPP1, SCP2, ERLIN2, HSD17B11, TRERF1, HSD17B6, NFYB, SRD5A3, CES1, FDX1, ELOVL6, ARV1* | DOI: 10.1186/s43042-019-0031-4 |
|  | Steroid hormone signaling pathway | *PPARGC1A, TGFB1I1, ESR1, CNOT2, DAB2, AR, MED12, KMT2D, CNOT1, GPER1, NCOA6, CARM1, TCF21, NEDD4, UBA5, UFL1, KDM3A, RNF4, FHL2, CTNNB1, MED17, TRIP4, CRY1, UFM1, ARNTL, CBFB, CALCOCO1, PHB, WBP2, MED24, KAT5, CALR, BRCA1, RNF14, OR51E2, UFSP2, MED30* |  |
|  | steroid hormone biosynthetic process | *SNAI2, NR1H4, GFI1, ERLIN2* |  |
| Androgen receptor signalling | Androgen receptor signaling pathway | *PPARGC1A, TGFB1I1, DAB2, AR, MED12, TCF21, KDM3A, RNF4, FHL2, CTNNB1, MED17, PHB, MED24, KAT5, BRCA1, RNF14, MED30* | PMID: 26306851 |
|  | Androgen receptor activity | *TGFB1I1, RACK1, GSN, PAWR, KAT2B, AR, REL, RXRB, FOXO1, PKN1, CTDSP1, NCOA6, CARM1, SRF, TGIF1, JUN, MAPK14, FKBP4, KDM3A, SMARCC1, FHL2, XRCC6, UBA3, CTNNB1, MAPK8, APPBP2, PRDX1, CDK6, KAT5, PIAS3, TCF4, PRKDC, XRCC5, BRCA1, SVIL, NR2C1* |  |
